# Potential effects of climate change on geographic distribution of the Tertiary relict tree species *Davidia involucrata* in China

**DOI:** 10.1038/srep43822

**Published:** 2017-03-08

**Authors:** Cindy Q. Tang, Yi-Fei Dong, Sonia Herrando-Moraira, Tetsuya Matsui, Haruka Ohashi, Long-Yuan He, Katsuhiro Nakao, Nobuyuki Tanaka, Mizuki Tomita, Xiao-Shuang Li, Hai-Zhong Yan, Ming-Chun Peng, Jun Hu, Ruo-Han Yang, Wang-Jun Li, Kai Yan, Xiuli Hou, Zhi-Ying Zhang, Jordi López-Pujol

**Affiliations:** 1 Institute of Ecology and Geobotany, Yunnan University, Kunming 650091, China; 2 Botanic Institute of Barcelona (IBB-CSIC-ICUB), Passeig del Migdia s/n, Barcelona 08038, Spain; 3 Center for International Partnerships and Research on Climate Change, Forestry and Forest Products Research Institute, Matsunosato 1, Tsukuba-shi, Ibaraki-ken, 305-8687, Japan; 4 Kunming Institute of Forestry Exploration and Design, the State Forestry Administration of China, Kunming 650216, China; 5 Kansai Research Center, Forestry and Forest Products Research Institute, Momoyama, Kyoto, 612-0855 Japan; 6 Tokyo University of Agriculture,1-1-1 Sakuragaoka, Setagaya-ku, Tokyo 156-8502, Japan; 7 Tokyo University of Information Sciences, 4-1 Onaridai Wakaba-ku, Chiba 265-8501, Japan; 8 Yunnan Academy of Forestry, Kunming 650204, China; 9 Key Laboratory of Mountain Ecological Restoration and Bioresource Utilization and Ecological Restoration Biodiversity Conservation, Key Laboratory of Sichuan Province, Chengdu Institute of Biology, Chinese Academy of Sciences, Chengdu 610041, China; 10 Center for Mountain Ecosystem Studies, Kunming Institute of Botany, Chinese Academy of Sciences, Kunming 650201, China; 11 Department of Life Science and Technology, Kunming University, Kunming 650214, China

## Abstract

This study, using species distribution modeling (involving a new approach that allows for uncertainty), predicts the distribution of climatically suitable areas prevailing during the mid-Holocene, the Last Glacial Maximum (LGM), and at present, and estimates the potential formation of new habitats in 2070 of the endangered and rare Tertiary relict tree *Davidia involucrata* Baill. The results regarding the mid-Holocene and the LGM demonstrate that south-central and southwestern China have been long-term stable refugia, and that the current distribution is limited to the prehistoric refugia. Given future distribution under six possible climate scenarios, only some parts of the current range of *D. involucrata* in the mid-high mountains of south-central and southwestern China would be maintained, while some shift west into higher mountains would occur. Our results show that the predicted suitable area offering high probability (0.5‒1) accounts for an average of only 29.2% among the models predicted for the future (2070), making *D. involucrata* highly vulnerable. We assess and propose priority protected areas in light of climate change. The information provided will also be relevant in planning conservation of other paleoendemic species having ecological traits and distribution ranges comparable to those of *D. involucrata*.

Since the maximum extension of ice sheets during LGM (Last Glacial Maximum, from 26,000 to 19,000 calendar years before the present, with cold conditions also prevailing until 14,500 yrs ago), the global climate has undergone rapid and remarkable changes toward a generally warmer current level^1^. To reduce the rate of species extinctions in a world dominated increasingly by human, natural protected areas are often the strategy on which conservation measures are built. However, in light of global climate change, conservation strategies and decisions regarding the location of protected areas must consider future regional climate changes and their effects on species distribution, including retractions and expansions of species ranges^2^,^3^,^4^. Determination of the extent of threatened plants’ response to climate change can be useful in formulating flexible conservation strategies for China^5^. Identifying past, contemporary and future climate refugia of Tertiary relict plants can clarify their conservation significance.

China enjoys great species diversity, with ca. 30,250 seed plant species. The key areas of south-central China and Indo-Burma (including most parts of Yunnan) are two of the 25 designated global biodiversity hotspots^6^. Two plant diversity centers with high levels of endemism are located in this region^7^. The area was never covered by extensive, unified ice-sheets, owing in part to its complex topography^8^ and to moderate cooling as compared to other regions of China^9^. Moreover, these areas did not suffer the extreme aridity that is usually associated with the glacial periods: within China, south-central and southwestern areas were those with the smallest LGM precipitation deficits, rainfall being even higher than at present during the autumn and winter seasons^9^. So the mountains of south-central and southwestern China have had a relatively stable long-term environment, and are now referred to as the Pleistocene glacial refugia^10^,^11^,^12^. Before and during the Ice Ages, a number of famous gymnosperms such as *Ginkgo, Metasequoia* and *Cathaya* disappeared from the rest of the Northern Hemisphere, and coincidentally, some temperate deciduous broad-leaved tree taxa died off in North America (e.g., *Davidia*^13^,^14^; *Tetracentron*^15^; *Cercidiphyllum* in review^16^), Europe (e.g., *Tetracentron*^17^; *Cercidiphyllum* in review^16^), the Russian Far East (e.g., *Tetracentron* in Kamchatka^18^), and Japan (e.g., *Davidia*^19^,^20^,^21^; *Tetracentron*^22^). Most of them now only survive in the humid subtropical areas of south-central and southwestern China. A number of these paleoendemics have highly restricted distributions, usually in the form of small, localized populations^23^. Refugia represent climatically stable areas and remain a high conservation priority as important sites for the long-term persistence of species. Today, they are in danger of overexploitation. Climate change has also become a great threat to these species and their forests.

*Davidia involucrata* Baill. (Figure 1) (dove tree or handkerchief tree in English, gongtong or gezishu in Chinese), included in Davidiaceae (by some in Nyssaceae or Cornaceae), is a rare and endangered tree species with small populations restricted to the humid mountains of south-central and southwestern China, mostly confined to the mountains surrounding the Sichuan Basin. The forests containing *D. involucrata* as one of the dominants are mainly scattered at 1300–1900 m altitude (but at 2300–2800 m in Yunnan) in areas with cool, foggy and cloudy climatic conditions. At these altitudes, *D. involucrata* co-dominates with other Tertiary relict plants including *Tetracentron, Cercidiphyllum, Tapiscia, Dipteronia, Pterostyrax, Carya, Liquidambar,* and *Decaisnea* (Fig. 2). *Davidia involucrata* also occurs, but very rarely, between 900–1300 m, growing with some evergreen broad-leaved trees of *Cyclobalanopsis, Castanopsis, Lithocarpus, Phoebe, Machilus* and *Neolitsea*. No adequate studies have been carried out to estimate the potential impact of climate change on this Tertiary relict species, and so far no conservation strategies that consider climate change have been proposed.

Species distribution modeling (SDM) combines species occurrence data with environmental variables, under the assumption that the distribution of known localities reflects survival patterns of species^24^. SDM has proven useful for estimating such patterns and the resultant risk of extinction. It can produce spatially explicit and comprehensive maps that are particularly valuable for identifying areas where conservation efforts and management strategies are most needed. Our main objective is to apply an SDM approach based on Tertiary relict tree species *D. involucrata*’s occurrence data and climatic surfaces at four different time points (LGM, mid-Holocene, present, and the year 2070), so as to estimate past distributions of climatically relevant areas, model the present potential distribution range, and predict potential distribution and vulnerability under future climate change. We hypothesize that the mountains of south-central and southwestern China have been long-term stable refugia of *D. involucrata* and that there would be a decline in areal extent of suitable habitats for this tree species under future climate change. In order to provide a basis for conservation management of *D. involucrata*, our aim is to map the past, current and future suitable habitat distributions of this paleoendemic species, and propose establishment of protected areas.

## Methods

### Study species and areas

*Davidia involucrata* is a deciduous broad-leaved tree and its fruit stone is heavy and woody, composed of fibers, each nut containing 3–6 seeds. It is a protected species in China, already included in the *National List of Rare and Endangered Plant Species* of 1984 (listed as “first grade” nationally protected) and later in the *Catalogue of the National Protected Key Wild Plants* of 1999 (also as “first grade”). Its relative rarity has also been recognized: the species was already included in the first red book of China (as a “rare” species)^25^, although in the red list of 2013—which follows the internationally recognized IUCN categories—the species is listed, surprisingly, as LC (“Least Concern”), that is, with no conservation concerns^26^. *Davidia involucrata* is scattered in isolated mountain slopes, or in valleys where the soil often contains much gravel, or by streams, or scree slopes harboring unique assemblages of plants^27^.

Today scattered stands of *D. involucrata* range approximately from 98–110°E, 26–32°N in south-central and southwestern China, including Hunan, Hubei, Chongqing, Sichuan, southern Shaanxi, southern Gansu, Guizhou and Yunnan Provinces. The latitudes are subtropical. Vegetation types in this area are diverse, according to altitudes, mainly including subtropical evergreen broad-leaved forests, warm temperate deciduous broad-leaved forests, deciduous and evergreen broad-leaved mixed forests, warm temperate coniferous forests, coniferous and broad-leaved mixed forests, cold-temperate coniferous forests, alpine scrub and meadow; however, by far most of the vegetation consists of what is commonly referred to as the subtropical evergreen broad-leaved forest. A striking feature is that within the transitional altitudinal zone from subtropical warm and humid evergreen to the temperate deciduous broad-leaved forests, where in some cases there is also an admixture of species of gymnosperms, a number of extant Tertiary relict plant species thrive today^23^. The climate is dominated by the Asian monsoon system, including the East Asian summer monsoon, the Indian summer monsoon, and the East Asian winter monsoon, with dry continental winds in winter and moist oceanic winds in summer. In general, in the subtropical areas, lands east of longitude 103° are more influenced by the East Asian summer monsoon, while to the west the Indian summer monsoon dominates.

### Data and distribution modeling

*Davidia involucrata*’s occurrence data for the whole of geographic distribution range were compiled from our extensive field survey (carried out within the area comprised between 26–32°N and 98–110°E), the Chinese Virtual Herbarium (CVH; http://www.cvh.org.cn/), and the Global Biodiversity Information Facility (GBIF; www.gbif.org/). Although many records from CVH did not specify latitude and longitude, we were able to geo-reference detailed locations, using only specimens with locations specified in the label information. In total, after removing duplicate records within each pixel (2.5 arc-min, *ca*. 5 km), we obtained 203 presence records.

A set of 19 bioclimatic variables at 2.5 arc-min resolution covering the distribution range (and neighboring areas) of *D. involucrata* under current conditions (*ca*. 1960–1990) were downloaded from the WorldClim website (www.worldclim.org)^28^. Although finer resolutions are available (30 arc-sec) for the bioclimatic variables, these may not be appropriate given uncertainties associated with geo-referencing approximate localities (or with geo-reference errors). We designed as the study area a region considerably larger than the current species range (15–56°N and 72–143°E). Although such an approach may tend to generalize conditions found near the occupied localities^29^, we have considered most of East Asia as the study area, given the complexity of this region in terms of topography and Neogene biogeographic history (including substantial marine transgressions/regressions) and the need to include also the areas where recent (Pliocene and Pleistocene) *Davidia* fossils have been found.

As the modeling algorithm, we employed the maximum entropy algorithm as implemented in MaxEnt v. 3.3 30, whose output is a map of habitat suitability ranging from 0 to 1 per grid cell. MaxEnt for species habitat modeling has some advantages compared to other SDM methods, including its convenience, its use of presence-only species records, and a generally excellent predictive performance^31^,^32^. We used only ‘linear’ and ‘quadratic’ feature to avoid producing too complex a model, which could lead to extrapolation errors^33^. The distribution model for *D. involucrata* under current conditions was projected to three time slices: mid-Holocene (ca. 6000 yr BP), LGM (ca. 21,000 yr BP), and 2070 (average for 2061–2080). For both past scenarios, we used paleoclimatic layers simulated by the Community Climate System Model Version 4 (CCSM4)^34^, the Model for Interdisciplinary Research on Climate Earth System Model (MIROC-ESM)^35^, and the New Earth System Model of the Max Planck Institute for Meteorology (MPI-ESM-P: http://www.mpimet.mpg.de/en/science/models/mpi-esm/). To predict potential shifts of the geographic distribution that might be caused by global climate change, we used three of the models with the best performance among the various available ones that have participated in the 5th Coupled Model Inter-Comparison Project (CMIP5) experiment^36^: CCSM4, the NOAA Geophysical Fluid Dynamics Laboratory Coupled Model 3 (GFDL-CM3)^37^, and MPI-ESM-LR. The three models were run in two of the four representative concentration pathways (RCPs) that were used in the Fifth Assessment IPCC report, RCP 2.6 and RCP 8.5 38. RCP 2.6 represents the most “benign” scenario (i.e., a likely increase of 0.3–1.7 °C for ca. 2081–2100), whereas RCP 8.5 is the most extreme scenario (a likely increase of 2.6–4.8 °C for ca. 2081–2100). All 19 bioclimatic variables for both past and future climate scenarios were also downloaded from the WorldClim website, at the same resolution (2.5 arc-min).

Selection of bioclimatic variables was done as follows: after a correlation analysis in 203 presence points and a random sample of 10000 pseudo-absence points within the study area (Supplementary Table S1), we grouped bioclimatic variables with strong collinearities (i.e., a Pearson’s correlation coefficient *r* ≥ |0.85|). Then, with all possible combinations of the 19 variables (but excluding those with more than two parameters from the same group of highly correlated variables) we calculated Variance Inflation Factors (VIF^39^); datasets with VIF >10 were excluded to avoid multi-collinearity. VIF were calculated using *vif* function of *usdm* package^40^ in R. The best combination among 6236 candidate parameter combinations was chosen following corrected Akaike Information Criterion (AICc^41^), and consisted of six variables: annual mean temperature (bio1), isothermality (bio3), temperature seasonality (bio4), precipitation seasonality (bio15), precipitation of the warmest quarter (bio18), and precipitation of the coldest quarter (bio19).

The “best” model was evaluated by 20-fold cross-validation with MaxEnt, with performance assessed using the area under the curve (AUC) of the receiver operating characteristic (ROC) plot. AUC scores may range between 0.5 (randomness) and 1 (exact match), with those above 0.9 indicating a good performance of the model^42^. The MaxEnt jackknife analysis was used to evaluate the relative importance of the six bioclimatic variables employed, based on their gain values when used in isolation. In *k*-fold cross-validation, occurrence data is divided randomly into *k* equal-size groups, and models are built using *k* − 1 bins for calibration in each iteration, with the left-out bin used for evaluation; background data are sampled by Maxent from the entire study region^43^. We applied the maximum sensitivity plus specificity (MSS) logistic threshold, which is very robust with all types of data^44^, to obtain a map of absence/presence (with the probability of presence shown as continuous values from the threshold to 1). The resulting map, which is the average of the multiple runs from the cross-validation, can be regarded as the “standard” model (Fig. 3A).

As suggested by many authors, uncertainty should be addressed in Ecological Niche Modeling (ENM) because it can produce large biases on the niche predictions^45^ and references therein. Thus, although our model performed well (AUC = 0.982 ± 0.006), a “consensus ensemble approach” or “consensus approach”^46^ was applied in order to visualize the variability in the predicted suitable areas among all generated models. A map showing such variability (or uncertainty) was obtained as follows (Fig. 3B): first all 20 continuous projection maps were converted into binary maps (applying the MSS threshold), and then were calculated how many models predict each pixel as a suitable area. Thus, a continuous map with pixels ranging from 0 to 20 was obtained. Converting these values to probabilities (i.e., 0 as 0%, 10 as 50%, or 20 as 100%), we finally obtained an estimate of each pixel’s probability of being predicted as suitable area. Every pixel for which at least 95% of the models (i.e., 19 of 20 and 20 of 20) forecast species presence was regarded as a pixel of high probability of being predicted by modeling (“presence pixel”). In an attempt to reduce the uncertainty in our models, those pixels not ranking as “presence pixels” were removed from the “standard” output maps to obtain a “refined” model where only those predicted areas with 95% of confidence are shown (Fig. 3C), and this procedure was applied for the present time and for all past and future scenarios (Supplementary Fig. S1). Although the areas predicted as suitable are somewhat reduced (for example, about 37% for the present time in *D. involucrata*; Fig. 3 and Supplementary Table S2) with this method, the robustness of the forecast is improved^46^. A proof of this is that the reduction of suitable area when the uncertainty is taken into account is mainly focused on those pixels with low logistic probability (threshold to 0.5, area loss = 46.54% for the present time; 0.5 to 1, area loss = 3.99 for the present time; Supplementary Table S2).

All ENM predictions were visualized in ArcGIS v. 10.2 (ESRI, Redlands, CA, USA). The suitable area (in km^2^) for all models at each time slice for *D. involucrata* was also calculated in ArcGIS. To estimate suitable area gains or losses (or unchanged areas) for both past and future scenarios with respect to the present, binary output maps were overlapped with the Intersect Tool of ArcGIS. To provide conservation advice on *D. involucrata*, the digitized map of Chinese protected areas was overlapped with the species occurrences and both binary maps of the present and future scenarios.

The climate data under various scenarios for the three time periods (present, mid-Holocene, LGM) are provided in the Supplementary Tables S3‒S4.

## Results

### Current distribution, model performance, and potential distribution under the present climate

The present distribution area of *D. involucrata* is confined to mountains with complex topographies surrounding the Sichuan Basin, the westernmost being in the Gaoligong Mountains of northwestern (NW) Yunnan, an extension of the southeastern Himalayas (Fig. 4A). Though *Davidia* fossils have been found dating from Pliocene to Early Pleistocene sediments in central Japan^19^,^20^,^21^, none have survived there (Fig. 4A).

We projected possible distribution of climatically suitable areas (potential habitats) for *D. involucrata* as expressed by occurrence probability (Fig. 4B). Our model showed excellent performance (AUC = 0.982 ± 0.006). Temperature seasonality (bio4), followed by the precipitation of the warmest quarter (bio18) and the annual mean temperature (bio1) (data not shown) were the most important variables determining the potential distribution of the species.

Present-day records are located in potential habitats under current climate (Fig. 4B). The area of potential habitats, after accounting for uncertainty, is 534,953 km^2^ using the threshold of 0.0644, but the habitats under high probability (0.5‒1) account for 34.5% of the total area of potential habitats (threshold‒1) (Table 1). Only a very few small areas in southern and central Japan appear as suitable but with low probability (<0.20) (although the suitable areas in Japan are much larger when uncertainty is not accounted; Supplementary Fig. S1A–C). The areas further south in Guizhou and SE Yunnan in China and further west (i.e., from Bhutan to Arunachal Pradesh in the East Himalayas) appear as potential habitats but *D. involucrata* is not found there at present (Fig. 4B). These so-called “empty habitats”^47^ are results of conditions other than climatic conditions.

### Potential distribution during the mid-Holocene (ca. 6,000 yr BP) and the LGM (ca. 21,000 yr BP)

We used scenarios mid-Holocene-CCSM, mid-Holocene-MIROC and mid-Holocene-MPI to model potential climatic habitats of *D. involucrata* during the mid-Holocene (*ca.* 6,000 yr BP). Under the three scenarios (Fig. 5), potential habitats were somewhat reduced (about 28.7% on average; Table 1), and were mainly located in Guizhou and the southern Sichuan Basin. The area further to the west (e.g., the East Himalayas) and the northern areas bordering the Sichuan Basin (except the mid-Holocene MPI) were, in contrast, generally not potential habitats during the mid-Holocene (although the East Himalayas appeared as potential when uncertainty is not taken into account: Supplementary Fig. S1D–L). Despite the loss on suitable areas, these were mostly included within the current potential habitats (78.5% on average; Fig. 5 and Table 1). Potential areas for the mid-Holocene not included in the current ones were limited to SE Yunnan (and NW Yunnan for the mid-Holocene-MPI; green areas in Fig. 5C,E and G).

We used scenarios LGM-CCSM, LGM-MIROC and LGM-MPI to model potential climatic habitats of *D. involucrata* during the LGM (*ca*. 21,000 yr BP). Under each of the three scenarios, the potential area increased to some extent (21.9% on average; Table 1) and, notably, overlap values were high (71.4% of the LGM with the present; 86.9% of the present with the LGM; Table 1). Area gains with respect to the present were mainly concentrated in the Sichuan Basin, whereas area losses occurred in the NW margin of the Sichuan Basin and the East Himalayas (Fig. 6C,E and G). Clearly the present-day potential distribution areas have been LGM refugia according to the three models (Fig. 6 and Table 1), including the East Himalayas (Fig. 6C and E).

### Potential distribution under future climate (2070)

Climate change effects on the geographic range of *D. involucrata* in 2070 are predicted under 2070-CCSM RCP 2.6, 2070-GFDL RCP 2.6, 2070-MPI RCP 2.6, 2070-CCSM RCP 8.5, 2070-GFDL RCP 8.5, and 2070-MPI RCP 8.5 scenarios (Figs 7 and 8).

Under the 2070-CCSM RCP 2.6 scenario, in south-central and southwestern China and the East Himalayas there would be potential habitats for *D. involucrata* (Fig. 7B), although the total areas would be somewhat reduced (32.2%), especially within the Sichuan Basin. In addition, the probability of suitable habitats in the northern area bordering the Sichuan Basin (i.e., SE Guansu) would be very low (<0.20) (Fig. 7B). Notably, most of the potential habitats are included within those now existing (92.3%; Fig. 7C and Table 1). The predicted suitable habitats with high probability (0.5‒1) would account for 37.3% of all the area of potential habitats (threshold‒1) (Table 1).

Under the 2070-GFDL RCP 2.6 scenario, the potential habitats would be considerably reduced (to 194,921 km^2^; that is, a loss of 63.6% of the present potential area; Table 1), being mainly limited to the western margins of the Sichuan Basin (Fig. 7D). Most of the potential habitats, however, are included within the present ones (76.3%); the non-overlapping areas of 2070-GFDL RCP 2.6 with the present would be limited to a stretch of land running from the NW Sichuan Basin to SE Gansu (that is, a slight northwestwards expansion), and to a lesser extent, in the East Himalayas (Fig. 7E). The suitable habitats having high probability (0.5‒1) would constitute only 20.7% of all the area of potential habitats (threshold‒1) (Table 1).

Under the 2070-MPI RCP 2.6 scenario, the predicted area of the potential habitat in 2070 would be reduced in a similar manner as in the 2070-CCSM RCP 2.6 model (Fig. 7F), with the sole exception of the East Himalayas (which do not appear as suitable in the MPI model). As for the MPI, most of the potential habitats are included within the ones for the present time (95.4%; Fig. 7G and Table 1), although the predicted suitable habitats with high probability (0.5‒1) would account for somewhat less (25.6%) of all the area of potential habitats (threshold‒1) (Table 1).

Under the 2070-CCSM RCP 8.5 scenario, the area of potential habitats of *Davidia involucrata* would be reduced (up to 59.1%; Fig. 8B and Table 1), and such losses would mainly occur in south-central China. In contrast, there would be a slight area extension in the western margin of its current potential habitats in southwestern China; in the East Himalayas, there would be both gains and losses of potential habitats. The area overlapping with the present potential habitats is still high (about 75%; Fig. 8C and Table 1). The suitable habitats with high probability (0.5–1) would account for only 36.5% of all the area of potential habitats (thresholds-1) (Table 1).

Comparing the potential habitats of *D. involucrata* under the 2070-GFDL RCP 8.5 scenario to the present climate, the loss would be critical (of almost 80%; Table 1). Potential habitats would be almost completely lost in south-central China, remaining almost exclusively in the west margins of the Sichuan Basin (Fig. 8D). The non-overlapping areas of 2070-GFDL RCP 8.5 with the present are almost the same as those for the RCP 2.6 scenario. For this case, however, the overlapping with the present is much lower (45.6%; Fig. 8E and Table 1). The suitable habitats having high probability (0.5‒1) would account for only 19.2% of all the area of potential habitats (threshold‒1) (Table 1).

Under the 2070-MPI RCP 8.5 scenario, the potential habitat would be largely reduced when compared to the present (73.6%; Table 1); suitable areas in south-central China would be greatly reduced, limited mostly to the margins of southwestern China. Moreover, the present habitat of *D. involucrata* in the East Himalayas would be totally lost (Fig. 8F). The area overlapping with the present potential habitats is still very high (83.5%), which means that the gained areas would be minimal (a very narrow strip at the NW margin of the present predicted area in southwestern China (Fig. 8G and Table 1). The suitable habitats with high probability (0.5‒1) would account for only 26.0% of all the area of potential habitats (threshold‒1) (Table 1).

In summary, under the six possible future climate scenarios, *D. involucrata* in south-central and southwestern China would suffer from substantial losses of potential habitats (with an average of nearly 57%), although the remaining areas are included within the current potential distribution area (Figs 7 and 8, Table 1). To a limited extent, and especially for the RCP 8.5 scenario, *D. involucrata* would shift further to the west (such a shift is much more evident when uncertainty is not taken into account; Supplementary Figs S1Ze–Zm). We must thus expect a decline in areal extent of suitable habitats for this species as global climate change progresses.

## Discussion

### Climate refugia

Tzedakis *et al*.^48^ define refugia as locations that provide habitats for the long-term persistence of populations. Thus, a location is classified as a refugium only if the supported population persists to the present. The refugium may represent the entire distribution of a species and be the last holdout before extinction, or be an isolated population that is disjunct from a more extensive distribution elsewhere. Refugia may be located within a former distribution as a result of a range contraction (*in situ* refugia or climate relicts); such populations persisting through several glacial cycles become long-term refugia^49^. Identifying and characterizing climate refugia provide an important context for understanding the development of modern distributions of species, traits and local adaptation^50^,^51^,^52^. Macrofossil data have shown *Davidia* existing in central Japan from Early Pliocene and Early Pleistocene sediments^19^,^20^,^21^; however, it went extinct in Japan probably during LGM (or in one of the previous glacial maxima). On the other hand, *Davidia* has persisted continuously in the mountains of south-central and southwestern China, making these areas into long-term refugia.

During the LGM, over four-fifths of the current potential distribution area would have been suitable for the persistence of the species, and even new areas would have been added as potential habitats (such as large areas within the Sichuan Basin); this “wealth” of potential habitats for *D. involucrata* during the LGM is likely related to the similar levels of precipitation (Bio12: annual precipitation; Bio18: precipitation of the warmest quarter) and more stable temperature (bio4: temperature seasonality) as compared to the present within the study area (Supplementary Fig. S2 and Tables S3 and S4). It should be noted here that not only other parts of China^9^, but also in most parts of Asia^53^ and the entire world^54^ the climate was much drier than at present, and it is generally assumed that availability of moisture is a prerequisite for the survival of species through the Pleistocene climatic cycles^55^,^56^,^57^. Indeed, the role of the ~3.5‒4 °C drop in LGM temperatures (Supplementary Fig. S2) would have been minimal regarding the *Davidia*’s potential habitats.

At the mid-Holocene, although the potential habitats for *D. involucrata* were somewhat reduced probably because of less stable temperature seasonality (bio4) (Supplementary Fig. S2) as compared to the present within the study area, these were mostly included within the current potential range (Fig. 5). Under future rapid climate warming, only part (Figs 7 and 8, Table 1) of the refugia will continue to have a suitable climate for this species; however, and as noted for the mid-Holocene, the suitable areas for the year 2070 are largely included within the present potential ones (and, notably, within high-probability ones; Figs 7 and 8). The potential areas of overlap between the current and future distribution of *D. involucrata* likely serve as *in situ* macrorefugia of this species from ongoing climate change. Given that no major changes are detected regarding the climatic variables more influential for *D. involucrata* except for bio1 (annual mean temperature), it seems clear that the expected rise of over 3 °C for the year 2070 would mean that the potential habitats for the species will be limited to the areas that show higher probability (>0.5) of maintaining the current climatic conditions. Only a few further western areas are identified as new climate refugia looking into the future (Figs 7 and 8). Although the 2070 models that do not take into account for uncertainty show a large expansion towards the west (to the much colder mountains of NW Sichuan, SE Gansu, SE Qinghai and SE Xizang; Supplementary Figs S1V‒Zm), the “refined” models only show limited range extensions, probably because most of these latter regions, despite being colder, are generally drier (now and in 2070) than the mountains of south-central and southwestern China (and, not suitable for harboring *D. involucrata* stands).

It can be concluded that past refugia of *D. involucrata* were restricted to the same locations as contemporary populations (that is, *in situ* survival, as usually reported for plant species in subtropical China^58^, especially the mountains in the Sichuan Basin and its adjacent areas^59^). This would probably be true again in the future, although sizes might well be smaller. Being aware of the extent and locations of the future climate refugia of *D. involucrata* in China and the East Himalayan region will help us to implement effective conservation management.

### Potential effects of future climate change on distribution

Climate model predictions for the future suppose a severe loss of biodiversity owing to changing climatic patterns^60^,^61^. Because of climate changes, species may no longer be adapted to the set of environmental conditions of a given region and may therefore fall outside its climatic niche^62^. Climate change will force some species to shift their geographic ranges, face extinction^63^,^64^. Species sensitive to temperature may respond to a warming climate by moving to cooler locations at higher latitudes or altitudes. Temperature seasonality, precipitation of the warmest quarter, and annual mean temperature would be the most effective factors regarding distribution of *D. involucrata*. A declining area of potential habitats for this species in 2070 indicates that *D. involucrata* will be greatly vulnerable under global climate change. With rapid warming, the potential habitats would dramatically shrink both in quantity (about 57%; Table 1) and quality [the high-probability (0.5‒1) habitats will shrank from 34.5% at present to an average of 27.5% for the year 2070; Table 1], while they would shift further west in southwestern China; however, this species has weak dispersal capacity because of its heavy stone fruits and seeds, and would have trouble migrating to new sites. Moreover, its regeneration mainly depends on sprouting from parent trees; seedling recruitments are poor^27^.

**Figure 2:**
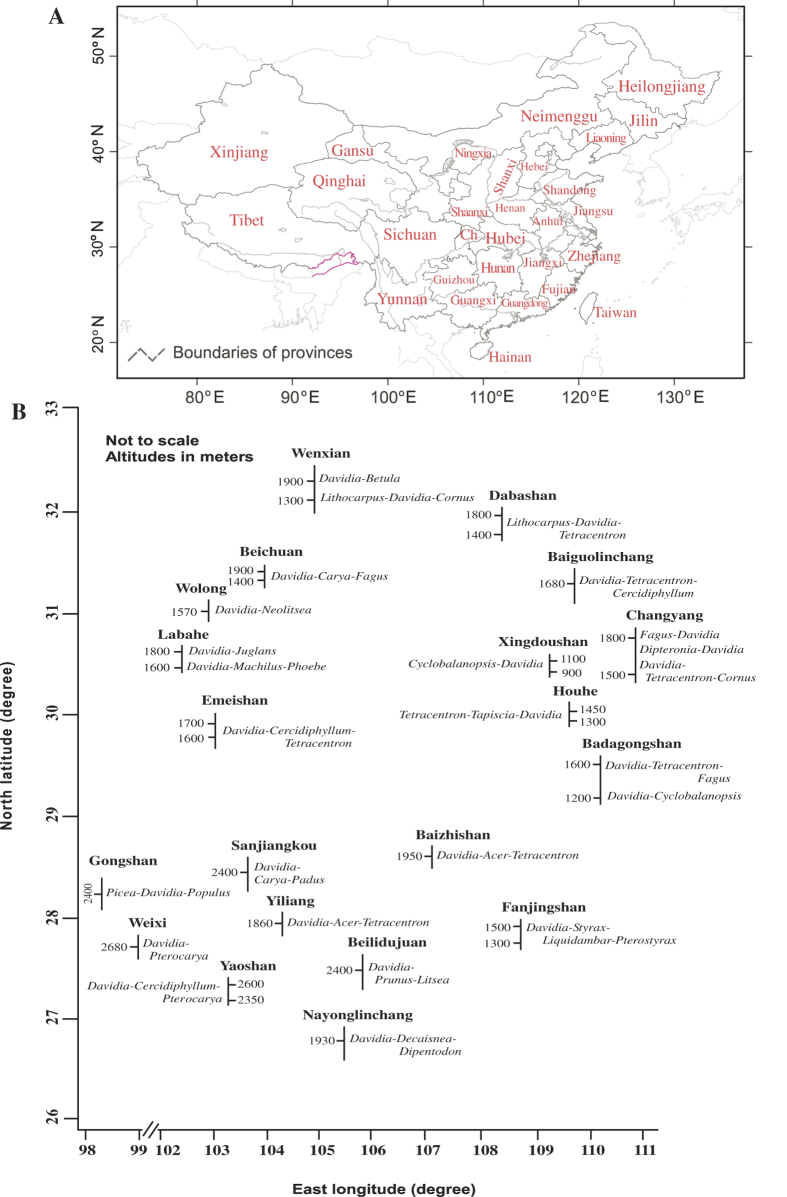
(**A**) Provinces of China. Ch = Chongqing. (**B**) The spatial distribution pattern of forests containing *D. involucrata* as one of the dominants. Purple lines: national boundaries between China and India (at issue). Maps were generated using the software ArcGIS v. 9.3.

**Figure 3:**
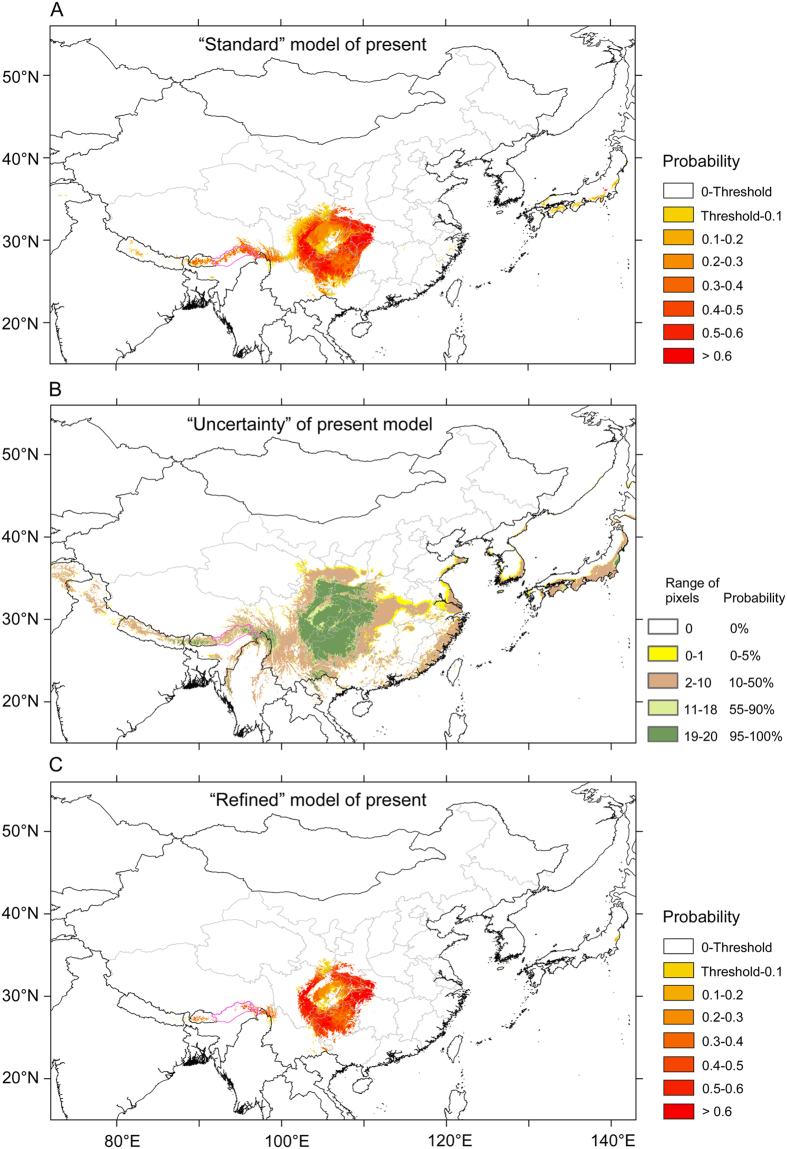
Schema of species distribution modeling in *Davidia involucrata*, taking into account the uncertainty derived from the evaluation of model performance. (**A**) A “standard” model for the present time period is obtained by averaging the multiple runs (20 in our case) from the cross-validation (or from another sampling technique, e.g. subsample); (**B**) a map showing the variability in the predicted suitable areas among all generated models (20) from the cross-validation (“uncertainty” map); (**C**) A “refined” map is obtained by removing the pixels that are forecast by <95% of the models (that is, the pixels shaded in yellow, brown and light green). Purple lines: national boundaries between China and India (at issue). Maps were generated using the software ArcGIS v. 9.3.

**Figure 4:**
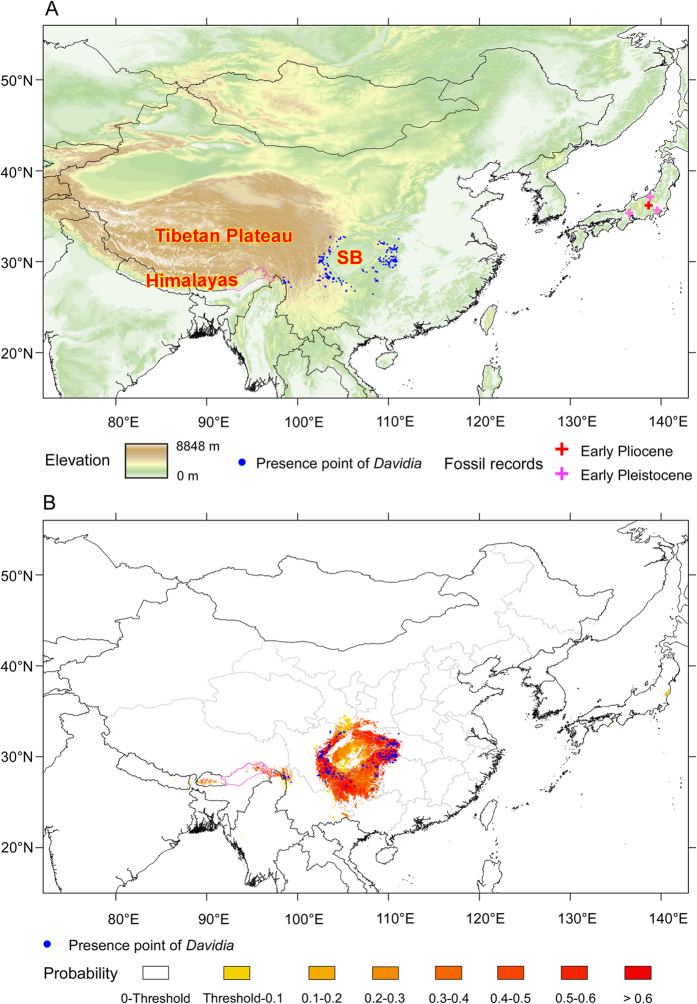
(**A**) Topography of the study area with *D. involucrata* occurrences. SB = Sichuan Basin. (**B**) Occurrence probabilities under present climate. Purple lines: national boundaries between China and India (at issue). Maps were generated using the software ArcGIS v. 10.2.

**Figure 5:**
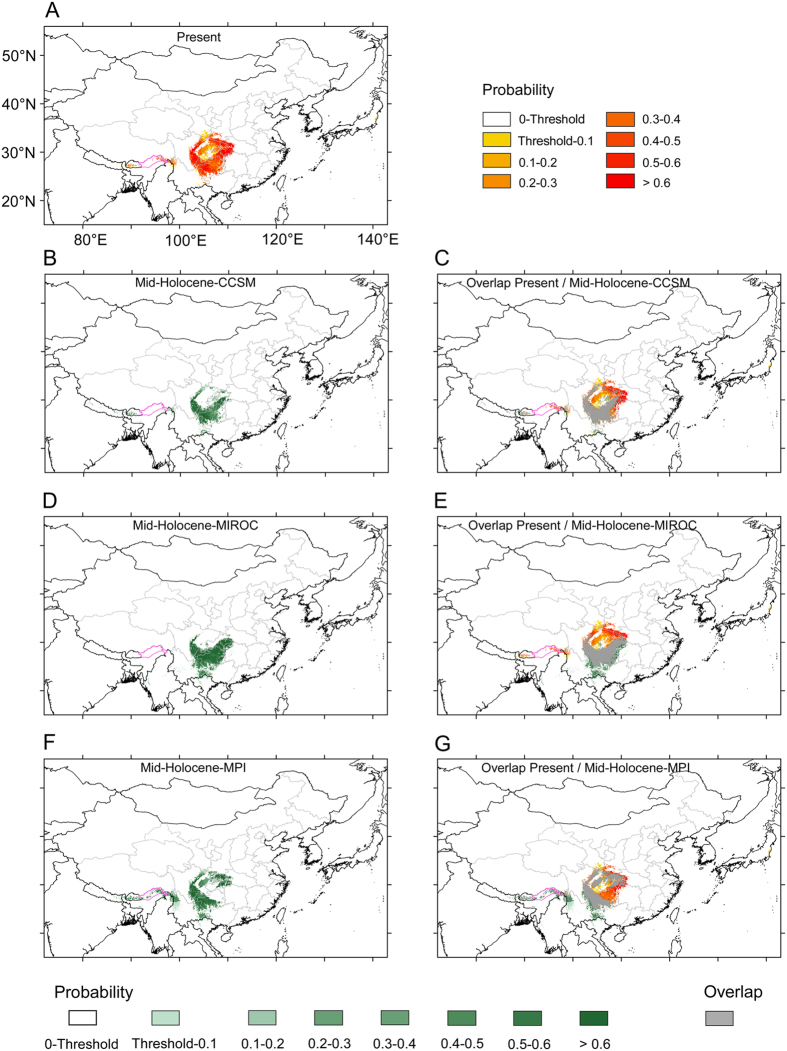
A comparison of potential habitats under the present climate and three climatic scenarios in the mid-Holocene. (**A**) Potential habitats under the present climate; (**B**) Potential habitats under the climatic scenario mid-Holocene-CCSM; (**C**) Overlap areas of the mid-Holocene-CCSM and the present. (**D**) Potential habitats under the climatic scenario mid-Holocene-MIROC; (**E**) Overlap areas of the mid-Holocene-MIROC and the present. (**F**) Potential habitats under the climatic scenario mid-Holocene-MPI. (**G**) Overlap areas of the mid-Holocene-MPI and the present. Purple lines: national boundaries between China and India (at issue). Maps were generated using the software ArcGIS v. 10.2.

**Figure 6:**
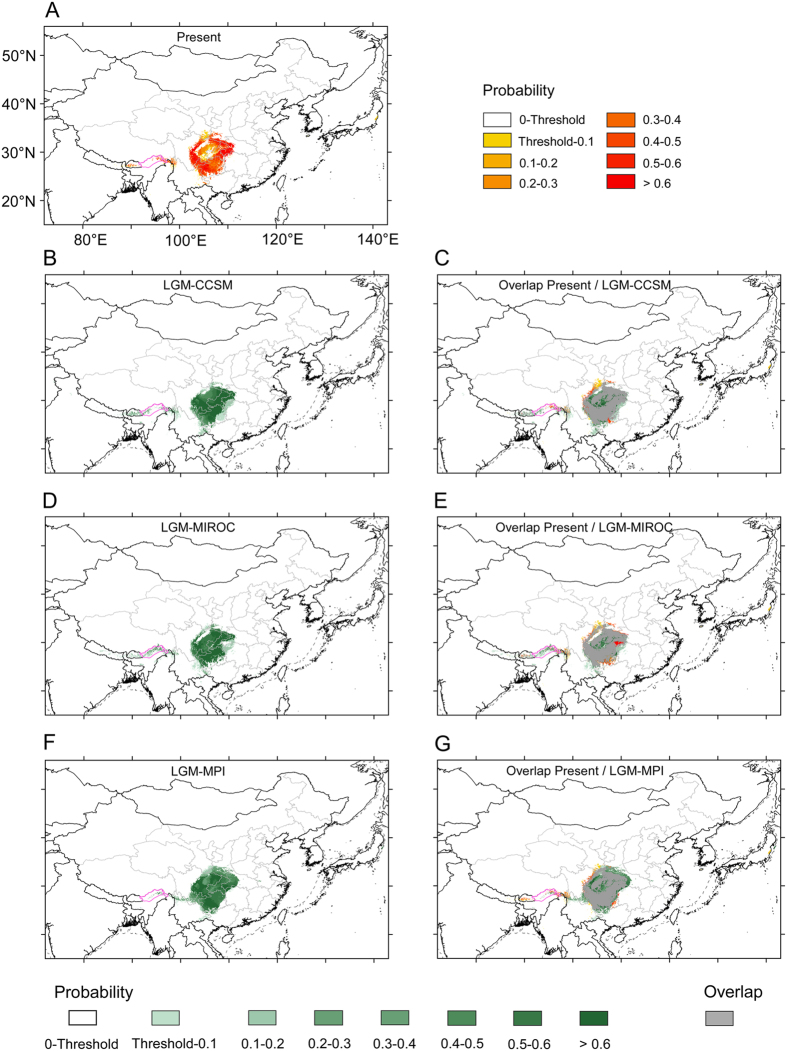
A comparison of potential habitats under the present climate and three climatic scenarios in the LGM. (**A**) Potential habitats under the present climate; (**B**) Potential habitats under the climatic scenario LGM-CCSM; (**C**) Overlap areas of the LGM-CCSM and the present. (**D**) Potential habitats under the climatic scenario LGM-MIROC; (**E**) Overlap areas of the LGM-MIROC and the present. (**F**) Potential habitats under the climatic scenario LGM-MPI. (**G**) Overlap areas of the LGM-MPI and the present. Purple lines: national boundaries between China and India (at issue). Maps were generated using the software ArcGIS v. 10.2.

**Figure 7:**
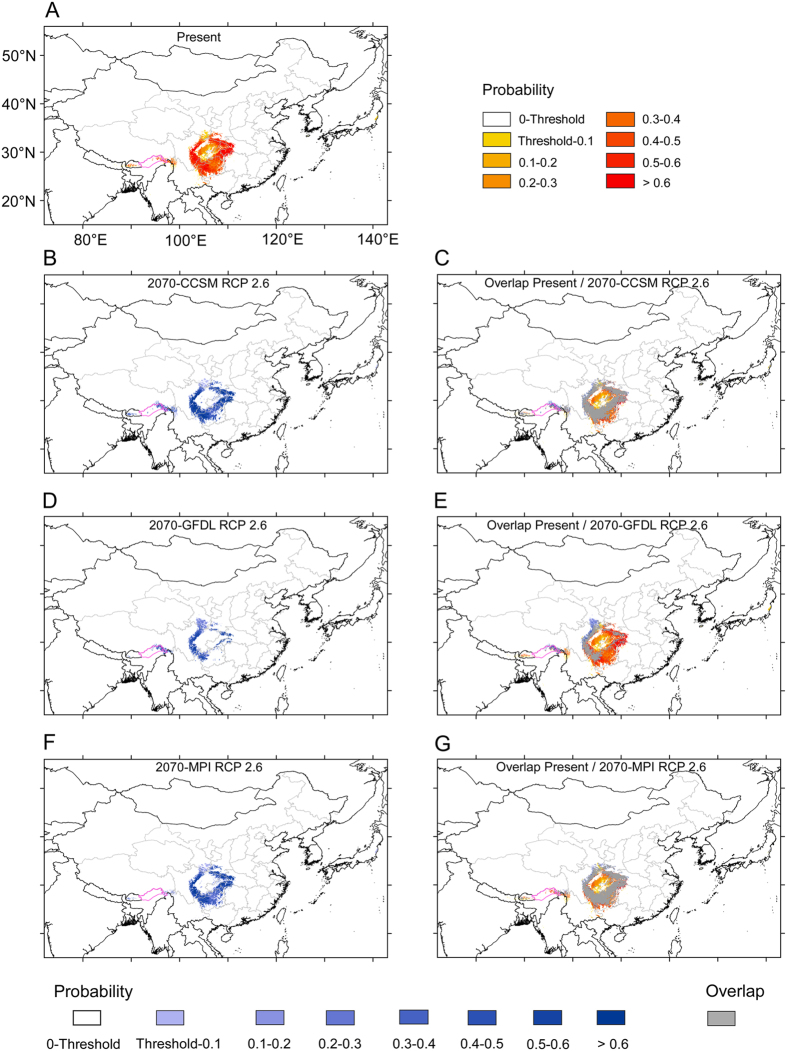
A comparison of potential habitats under the present climate and three climatic scenarios in future (2070). (**A**) Potential habitats under the present climate; (**B**) Potential habitats under the climatic scenario 2070-CCSM RCP 2.6; (**C**) Overlap areas of the 2070-CCSM RCP 2.6 and the present; (**D**) Potential habitats under the climatic scenario 2070-GFDL RCP 2.6; (**E**) Overlap areas of the 2070-GFDL RCP 2.6 and the present; (**F**) Potential habitats under the climatic scenario 2070-MPI RCP 2.6; (**G**) Overlap areas of the 2070-MPI RCP 2.6 and the present. Purple lines: national boundaries between China and India (at issue). Maps were generated using the software ArcGIS v.10.2.

**Figure 8:**
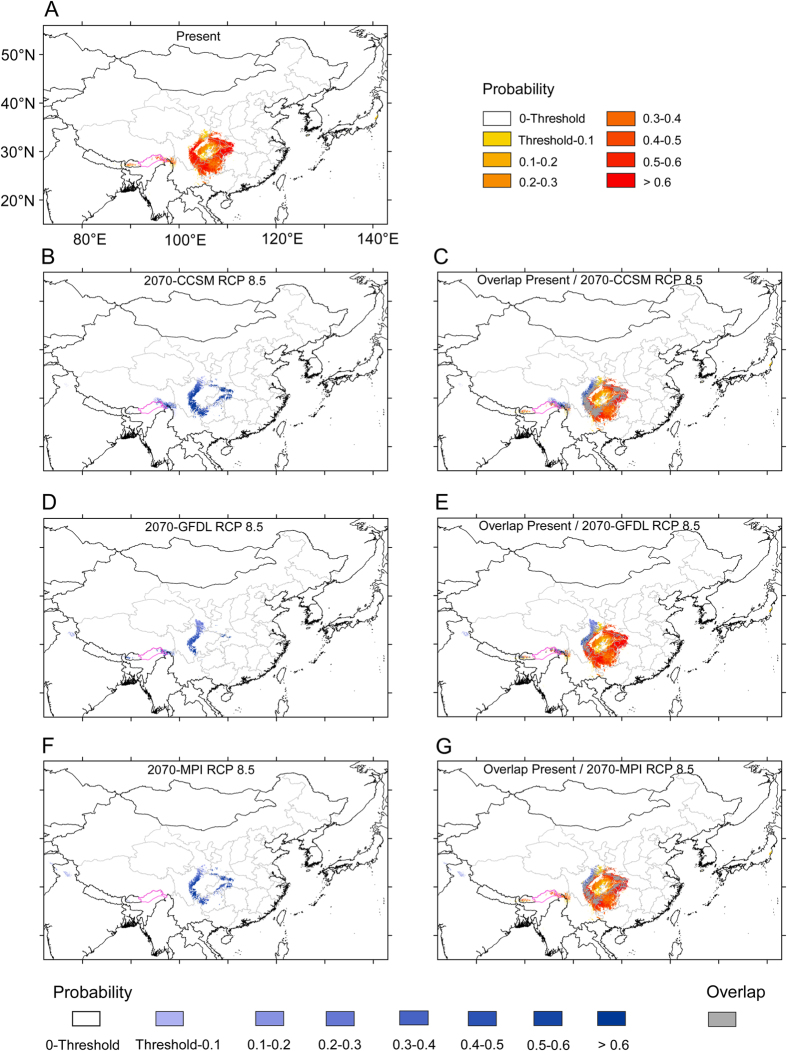
A comparison of potential habitats under the present climate and three climatic scenarios in future (2070). (**A**) Potential habitats under the present climate; (**B**) Potential habitats under the climatic scenario 2070-CCSM RCP 8.5; (**C**) Overlap areas of the 2070-CCSM RCP 8.5 and the present; (**D**) Potential habitats under the climatic scenario 2070-GFDL RCP 8.5; (**E**) Overlap areas of the 2070-GFDL RCP 8.5 and the present; (**F**) Potential habitats under the climatic scenario 2070-MPI RCP 8.5; (**G**) Overlap areas of the 2070-MPI RCP 8.5 and the present. Purple lines: national boundaries between China and India (at issue). Maps were generated using the software ArcGIS v. 10.2.

Many plants are considered ecological conservatives; they cannot adapt themselves sufficiently to changing climatic conditions^65^,^66^. Svenning^67^ found strong evolutionary conservatism in climatic requirements among the temperate tree floras of Europe, Asia and North America. *Davidia involucrata* grows with other paleoendemic temperate deciduous tree species, including *Tetracentron sinense, Cercidiphyllum japonicum, Tapiscia sinensis, Dipteronia sinensis, Pterostyrax psilophyllus, Carya cathayensis, Liquidambar acalycina*, and *Decaisnea insignis* (Fig. 2) in specific topographic habitats in the cloudy and foggy forest zones of certain altitudes, as on scree slopes where competition from other plants is rather limited^27^. All the paleoendemic species are habitat specialists. Niche evolution might be impossible for these Tertiary relict trees. They cannot evolve significantly new adaptations or disperse to new localities in the time projected for climate change, nor can they compete with non-paleoendemic evergreen broad-leaved species. Clearly, *D. involucrata* and other paleoendemic tree species growing in the same plant communities must be threatened by future climate warming.

### Conservation management: recommendations

**Figure 9:**
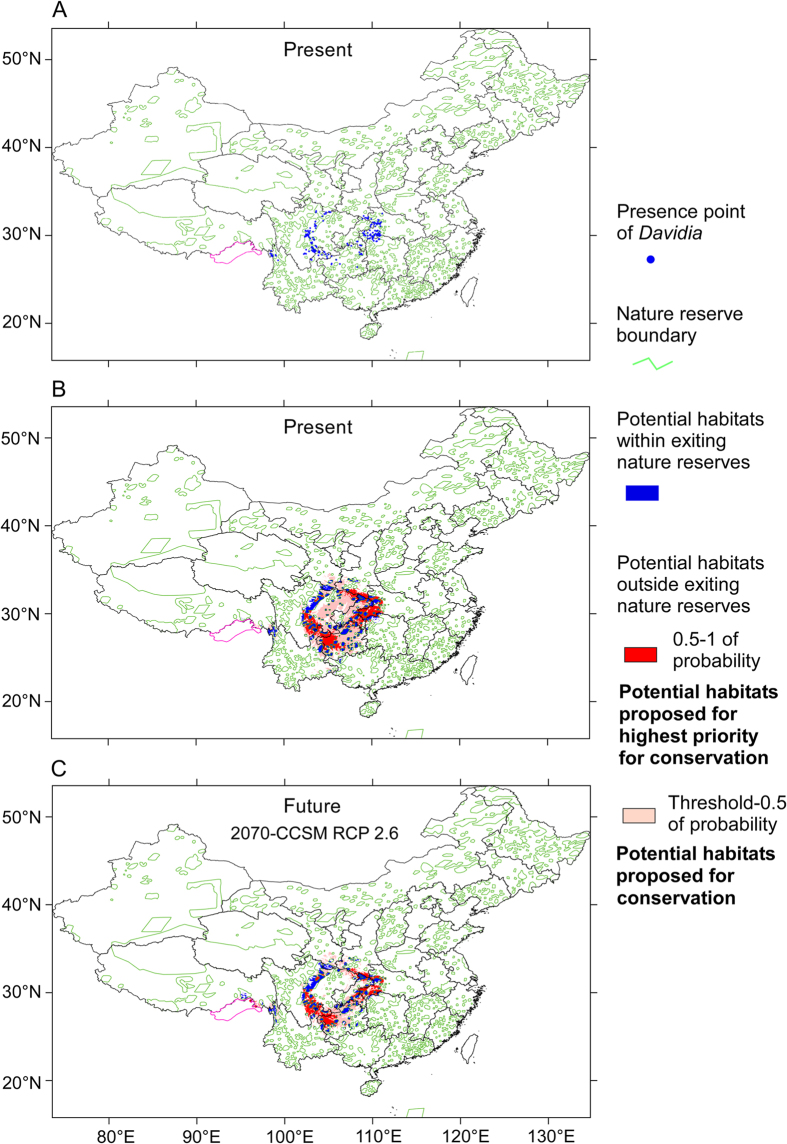
(**A**) Current presence points of *D. involucrata* within and outside existing protected areas (nature reserves) of China. (**B**) Modeled potential habitats with and without protection under the present climate in China, with areas proposed for conservation. (**C**) Predicted potential habitats with and without protection under a future 2070 climate in China, with areas proposed for conservation, exemplified by the scenario 2070-CCSM RCP 2.6. Purple lines: national boundaries between China and India (at issue). Maps were generated using the software ArcGIS v. 10.2.

**Figure 10:**
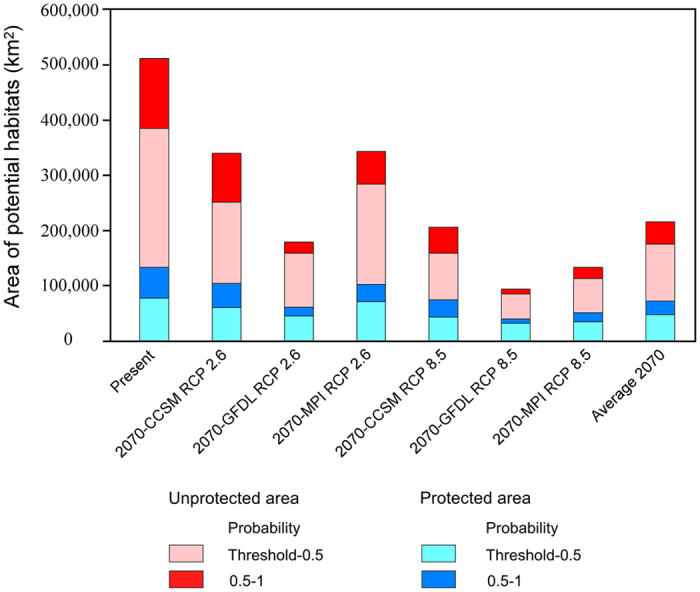
Area of potential habitats of *D. involucrata* with and without protection in China.

Because of the complicated policies of nature reserve management in different countries, and because the current distribution range of *D. involucrata* is only in China, also because 94% of future (2070) potential distribution range of this species is within China, we only discuss conservation strategies in China in this study. Climate change may threaten habitat suitability of threatened plant species within Chinese nature reserves^68^. Establishment and management of protected areas (nature reserves) must include consideration of all the current distributed sites of *D. involucrata*, and must recognize the species’ future distribution in response to future climate conditions. Up to 56.3% of the present-day presence points of this species are outside the protected areas (nature reserves) of China (Fig. 9A). Under the present climate, 73.9% of modeled potential habitats are outside the existing protected areas (nature reserves) (Figs 9B and 10, and Supplementary Table S5). Under future 2070-CCSM RCP 2.6 scenario, 69.3% of potential habitats in China would be outside the protected areas (nature reserves) (Figs 9C and 10, and Supplementary Table S5). Under the other five scenarios for the year 2070, 57.6‒70.2% of potential habitats in China would be outside the protected areas (Fig. 10 and Supplementary Fig. S3 and Table S5). On an average of all the six predicted models for the year 2070, 64.8% potential habitats in China would be outside the network of nature reserves (Figs 9C and 10, and Supplementary Fig. S3 and Table S5). In view of the effects of global climate change on this species’ geographic distribution, we urge designation of these currently unprotected areas as priority for conservation, and particularly the establishment of the highest priority protected areas for the most suitable habitats (i.e., the high-probability ones, 0.5‒1). As seen in Fig. 9 and Supplementary Fig. S3, the most urgent needs for new nature reserves are in the south-western mountainous margin of the Sichuan Basin and the central China Mountains (that is, around the Three Gorges Region). In addition, despite not harboring highly suitable habitats (threshold‒0.5), (i.e., Dalou Mountains), it is urgent to set up or enlarge the current protected areas in the southeastern mountainous margin of the Sichuan Basin, in order to maintain the current genetic corridor between the eastern and western lineages of *D. involucrata*^69^. At present this area is the only one that effectively connects genetically both lineages (such a corridor does not exist along the northern margins of the Sichuan Basin^69^). As an additional measure, introduction/reintroduction of *Davidia* stands would be an efficient measure to avoid the growing physical isolation of the current patches (and, thus, offsetting the effects of genetic isolation). We should bear in mind that the species is prone to genetic isolation because of its proven weak seed dispersal capacity^69^, a probably low pollen dispersal ability^70^, a poor germination^71^ and low seedling recruitment rate^27^. The combination of distributional, genetic, and biological information can be used to conserve *D. involucrata* and nearby Tertiary relict species through effective and efficient management planning in the Nature Reserve system and in other management activities (e.g., translocations).

Besides planning and establishing the protected areas, we further propose to monitor population size and demographic structure of *D. involucrata* and conserve the species *in situ*, and to implement propagation of the species outside its natural range (as artificial stands in experimental plots), to enhance its chances for long-term survival.

## Additional Information

**How to cite this article:** Tang, C. Q. *et al*. Potential effects of climate change on geographic distribution of the Tertiary relict tree species *Davidia involucrata* in China. *Sci. Rep.* **7**, 43822; doi: 10.1038/srep43822 (2017).

**Publisher's note:** Springer Nature remains neutral with regard to jurisdictional claims in published maps and institutional affiliations.

## Supplementary Material

Supplementary Information

## Figures and Tables

**Figure 1:**
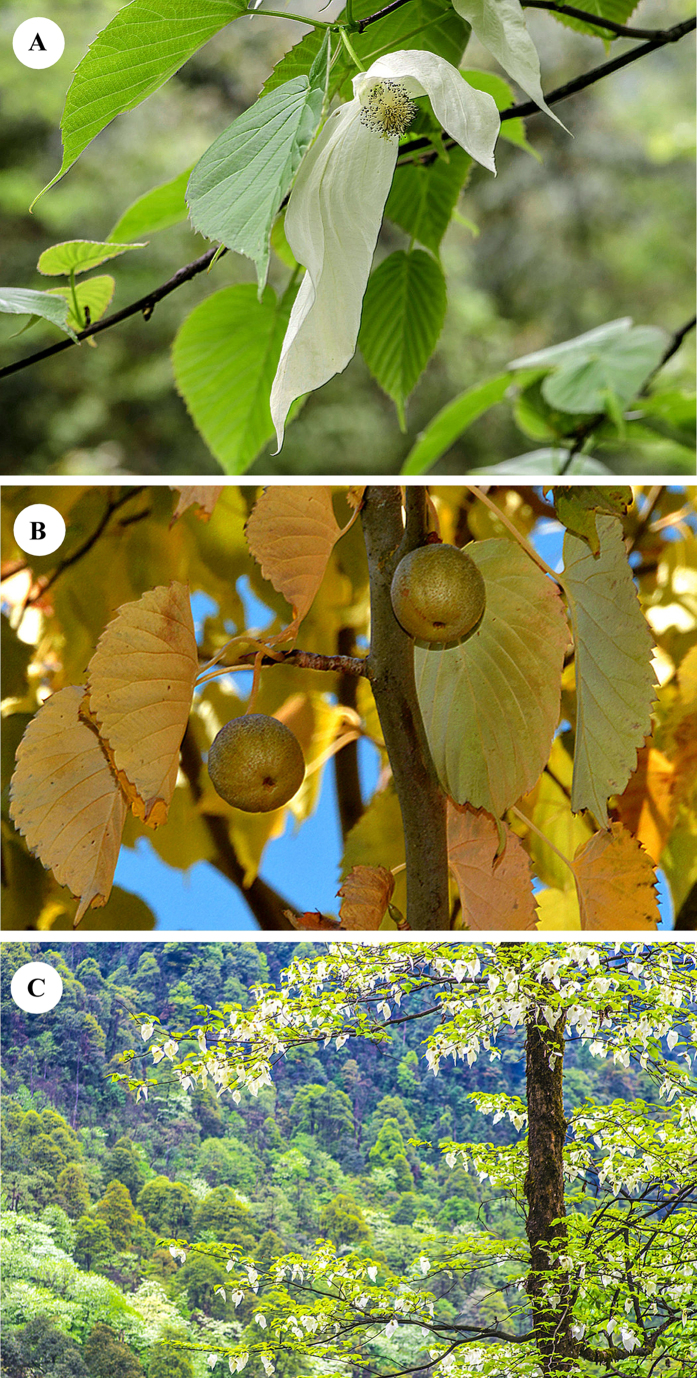
(**A**) An inflorescence with two showy white bracts of *D. involucrata* (Photograph by Shi-Liu Wang). (**B**) Fresh fruits with autumn foliage *D. involucrata* (Photograph by Cindy Q. Tang). (**C**) A forest of *D. involucrata* at ca. 1480 m in Longcanggou, Sichuan (Photograph by Jun Hu).

**Table 1:** Predicted potential distribution of *Davidia involucrata*.

Model	Total Predicted area (km^2^)	Predicted area (km^2^) from threshold–0.5	Predicted area (km^2^) from 0.5–1	Difference with respect to the present (km^2^ and %)	Overlap with the present (km^2^ and %)	Overlap of the present with each model (%)
Present	534,953	350,488	184,465	—	—	
Mid-Holocene-CCSM	292,256	131,119	161,137	242,697 (−45.37)	271,543 (92.91)	50.76
Mid-Holocene-MIROC	408,637	116,960	291,677	126,316 (−23.61)	293,095 (71.73)	54.79
Mid-Holocene-MPI	444,012	202,967	241,045	90,941 (−17.00)	313,977 (70.71)	58.69
*Average Holocene*	*381,635*	*150,349*	*231,286*	*153,318 (*−*28.66)*	*292,872 (78.45)*	*54.75*
LGM-CCSM	654,131	283,554	370,577	119,178 (+22.28)	471,444 (72.07)	88.13
LGM-MIROC	605,354	215,318	390,036	70,401 (+13.16)	446,446 (73.75)	83.46
LGM-MPI	696,462	287,786	408,676	161,509 (+30.19)	476,104 (68.36)	89.00
*Average LGM*	*651,982*	*262,219*	*389,763*	*117,029 (+21.88)*	*464,665 (71.39)*	*86.86*
2070-CCSM RCP 2.6	362,542	227,309	135,233	172,411 (−32.23)	334,527 (92.27)	62.53
2070-GFDL RCP 2.6	194,921	154,649	40,272	340,032 (−63.56)	148,629 (76.25)	27.78
2070-MPI RCP 2.6	353,095	262,546	90,549	181,858 (−34.00)	336,952 (95.43)	62.99
2070-CCSM RCP 8.5	218,920	138,977	79,943	316,033 (−59.08)	164,029 (74.93)	30.66
2070-GFDL RCP 8.5	110,608	89,413	21,195	424,345 (−79.32)	50,406 (45.57)	9.42
2070-MPI RCP 8.5	141,457	104,742	36,715	393,496 (−73.56)	118,065 (83.46)	22.07
*Average 2070*	*230,257*	*162,939*	*67,318*	*304,696 (*−*56.96)*	*192,101 (77.99)*	*35.91*
