# Supplementary material for: Potential effects of climate change on geographic distribution of the Tertiary relict tree species Davidia involucrata in China
Source: Sci Rep. 2017 Mar 8;7:43822. doi: 10.1038/srep43822 (PMC5341038; doi:10.1038/srep43822)

Supplementary information

Potential effects of climate change on geographic distribution of the Tertiary relict tree species *Davidia involucrata* in China

Cindy Q. Tang[1*], Yi-Fei Dong[1], Sonia Herrando-Moraira[2], Tetsuya Matsui[3], Haruka Ohashi[3], Long-Yuan He[4], Katsuhiro Nakao[5], Nobuyuki Tanaka[6], Mizuki Tomita[7], Xiao-Shuang Li[8], Hai-Zhong Yan[1], Ming-Chun Peng[1*], Jun Hu[9], Ruo-Han Yang[1], Wang-Jun Li[1], Kai Yan[10], Xiuli Hou[11], Zhi-Ying Zhang[1], Jordi López-Pujol[2]

[1] Institute of Ecology and Geobotany, Yunnan University, Kunming 650091, China
[2] Botanic Institute of Barcelona (IBB-CSIC-ICUB), Passeig del Migdia s/n, Barcelona 08038, Spain
[3] Center for International Partnerships and Research on Climate Change, Forestry and Forest Products Research Institute, Matsunosato 1, Tsukuba-shi, Ibaraki-ken, 305-8687, Japan
[4] Kunming Institute of Forestry Exploration and Design, the State Forestry Administration of China, Kunming 650216, China
[5] Kansai Research Center, Forestry and Forest Products Research Institute, Momoyama, Kyoto, 612-0855 Japan
[6] Tokyo University of Agriculture, 1-1-1 Sakuragaoka, Setagaya-ku, Tokyo 156-8502, Japan
[7] Tokyo University of Information Sciences, 4-1 Onaridai Wakaba-ku, Chiba 265-8501, Japan
[8] Yunnan Academy of Forestry, 650204 Kunming, China
[9] Key Laboratory of Mountain Ecological Restoration and Bioresource Utilization and Ecological Restoration Biodiversity Conservation, Key Laboratory of Sichuan Province, Chengdu Institute of Biology, Chinese Academy of Sciences, Chengdu 610041, China
[10] Center for Mountain Ecosystem Studies, Kunming Institute of Botany, Chinese Academy of Sciences, 650201 Kunming, China
[11] Department of Life Science and Technology, Kunming University, Kunming 650214, China

Correspondence and request for materials should be addressed to C.Q.T (e-mail: cindyqtang@aol.com) or M.-C.P. (email: mchpeng@ynu.edu.cn)

Table S1. Pearson's correlations among the 19 bioclimatic variables used in this study (underlined, coefficient values ≥ |0.85|). The meaning of all variables is included in the footnote (in bold are the selected variables for building the SDM definitive models).

	bio1	bio2	bio3	bio4	bio5	bio6	bio7	bio8	bio9	bio10	bio11	bio12	bio13	bio14	bio15	bio16	bio17	bio18	bio19
bio1	1	-0.401	0.607	-0.765	0.853	0.967	-0.751	0.833	0.941	0.905	0.970	0.638	0.646	0.181	0.122	0.652	0.241	0.385	0.258
bio2	-0.401	1	0.061	0.375	-0.185	-0.496	0.547	-0.254	-0.384	-0.322	-0.417	-0.646	-0.469	-0.636	0.526	-0.493	-0.663	-0.554	-0.619
bio3	0.607	0.061	1	-0.857	0.254	0.687	-0.760	0.281	0.689	0.284	0.732	0.447	0.519	-0.178	0.384	0.523	-0.111	0.278	-0.048
bio4	-0.765	0.375	-0.857	1	-0.339	-0.889	0.977	-0.362	-0.851	-0.420	-0.897	-0.639	-0.624	-0.139	-0.134	-0.640	-0.207	-0.427	-0.238
bio5	0.853	-0.185	0.254	-0.339	1	0.704	-0.306	0.908	0.719	0.983	0.712	0.362	0.409	0.087	0.130	0.402	0.119	0.108	0.133
bio6	0.967	-0.496	0.687	-0.889	0.704	1	-0.891	0.701	0.960	0.778	0.995	0.697	0.677	0.243	0.064	0.688	0.308	0.441	0.327
bio7	-0.751	0.547	-0.760	0.977	-0.306	-0.891	1	-0.359	-0.828	-0.416	-0.880	-0.704	-0.646	-0.270	-0.003	-0.666	-0.336	-0.522	-0.353
bio8	0.833	-0.254	0.281	-0.362	0.908	0.701	-0.359	1	0.635	0.934	0.705	0.452	0.481	0.107	0.172	0.477	0.138	0.308	0.108
bio9	0.941	-0.384	0.689	-0.851	0.719	0.960	-0.828	0.635	1	0.768	0.964	0.611	0.613	0.175	0.077	0.621	0.244	0.334	0.291
bio10	0.905	-0.322	0.284	-0.420	0.983	0.778	-0.416	0.934	0.768	1	0.777	0.473	0.493	0.173	0.084	0.490	0.211	0.245	0.214
bio11	0.970	-0.417	0.732	-0.897	0.712	0.995	-0.880	0.705	0.964	0.777	1	0.675	0.673	0.182	0.130	0.683	0.248	0.418	0.272
bio12	0.638	-0.646	0.447	-0.639	0.362	0.697	-0.704	0.452	0.611	0.473	0.675	1	0.932	0.480	-0.079	0.953	0.544	0.834	0.520
bio13	0.646	-0.469	0.519	-0.624	0.409	0.677	-0.646	0.481	0.613	0.493	0.673	0.932	1	0.232	0.183	0.992	0.300	0.746	0.298
bio14	0.181	-0.636	-0.178	-0.139	0.087	0.243	-0.270	0.107	0.175	0.173	0.182	0.480	0.232	1	-0.580	0.250	0.985	0.392	0.927
bio15	0.122	0.526	0.384	-0.134	0.130	0.064	-0.003	0.172	0.077	0.084	0.130	-0.079	0.183	-0.580	1	0.148	-0.565	-0.080	-0.525
bio16	0.652	-0.493	0.523	-0.640	0.402	0.688	-0.666	0.477	0.621	0.490	0.683	0.953	0.992	0.250	0.148	1	0.318	0.771	0.312
bio17	0.241	-0.663	-0.111	-0.207	0.119	0.308	-0.336	0.138	0.244	0.211	0.248	0.544	0.300	0.985	-0.565	0.318	1	0.444	0.952
bio18	0.385	-0.554	0.278	-0.427	0.108	0.441	-0.522	0.308	0.334	0.245	0.418	0.834	0.746	0.392	-0.080	0.771	0.444	1	0.378
bio19	0.258	-0.619	-0.048	-0.238	0.133	0.327	-0.353	0.108	0.291	0.214	0.272	0.520	0.298	0.927	-0.525	0.312	0.952	0.378	1

bio1 = annual mean temperature; **bio2 = mean diurnal range [mean of monthly (max temp − min temp)]**; bio3 = isothermality [(bio2/bio7) × 100]; **bio4 = temperature seasonality (standard deviation × 100)**; bio5 = maximum temperature of the warmest month; bio6 = minimum temperature of the coldest month; bio7 = temperature annual range (bio5 − bio6); bio8 = mean temperature of the wettest quarter; **bio9 = mean temperature of the driest quarter**; **bio10 = mean temperature of the warmest quarter**; bio11 = mean temperature of the coldest quarter; bio12 = annual precipitation; bio13 = precipitation of the wettest month; bio14 = precipitation of the driest month; **bio15 = precipitation seasonality**; **bio16 = precipitation of the wettest quarter**; bio17 = precipitation of the driest quarter; **bio18 = precipitation of the warmest quarter**; **bio19 = precipitation of the coldest quarter**.

Table S2. Reduction of suitable area for *Davidia involucrata* after uncertainty is taking into account.

	**"Standard" models**			**"Refined" models**			**Percentage of area loss applying uncertainties (%)**		
**Model**	**Total Predicted area (km^2)**	**Predicted area (km^2) from threshold–0.5**	**Predicted area (km^2) from 0.5–1**	**Total Predicted area (km^2)**	**Predicted area (km^2) from threshold–0.5**	**Predicted area (km^2) from 0.5–1**	**Total Predicted area (km^2)**	**Predicted area (km^2) from threshold–0.5**	**Predicted area (km^2) from 0.5–1**
Present	847,764	655,617	192,147	534,953	350,488	184,465	36.9	46.54	3.99
Mid-Holocene-CCSM	873,910	699,223	174,687	292,256	131,119	161,137	66.56	81.25	7.76
Mid-Holocene-MIROC	945,504	606,970	338,534	408,637	116,960	291,677	56.78	80.73	13.84
Mid-Holocene-MPI	871,248	610,243	261,005	444,012	202,967	241,045	49.04	66.74	7.65
Average Holocene	*896,887*	*638,812*	*258,075*	*381,635*	*150,349*	*231,286*	*57.46*	*76.24*	*9.75*
LGM-CCSM	984,920	606,938	377,982	654,131	283,554	370,577	33.59	53.28	1.96
LGM-MIROC	1,102,804	651,027	451,777	605,354	215,318	390,036	45.11	66.93	13.67
LGM-MPI	903,715	492,204	411,511	696,462	287,786	408,676	22.93	41.53	0.69
Average LGM	*997,146*	*583,390*	*413,757*	*651,982*	*262,219*	*389,763*	*33.88*	*53.91*	*5.44*
2070-CCSM RCP 2.6	839,830	698,690	141,140	362,542	227,309	135,233	56.83	67.47	4.19
2070-GFDL RCP 2.6	847,500	799,674	47,826	194,921	154,649	40,272	77.00	80.66	15.79
2070-MPI RCP 2.6	715,079	624,053	91,026	353,095	262,546	90,549	50.62	57.93	0.52
2070-CCSM RCP 8.5	733,917	650,827	83,090	218,920	138,977	79,943	70.17	78.65	3.79
2070-GFDL RCP 8.5	878,918	847,573	31,345	110,608	89,413	21,195	87.42	89.45	32.38
2070-MPI RCP 8.5	521,571	484,006	37,565	141,457	104,742	36,715	72.88	78.36	2.26
Average 2070	*756,136*	*684,137*	*71,999*	*230,257*	*162,939*	*67,318*	*69.15*	*75.42*	*9.82*

Table S3. Mean values for the 19 bioclimatic variables for the study area (i.e. the rectangle comprised between 15–56°N and 72–143°E). The mean values have been obtained averaging the individual values for all the 2.5 arc-min cells contained within the study area 1,239,717 cells).

	**Variable**																		
**Climatic scenario**	bio1 (ºC)	bio2 (ºC)	bio3 (%)	bio4 (ºC × 100)	bio5 (ºC)	bio6 (ºC)	bio7 (ºC)	bio8 (ºC)	bio9 (ºC)	bio10 (ºC)	bio11 (ºC)	bio12 (mm)	bio13 (mm)	bio14 (mm)	bio15 (%)	bio16 (mm)	bio17 (mm)	bio18 (mm)	bio19 (mm)
Present	7.15±10.62	11.99±2.23	31.01±9.20	1012.20±413.41	26.60±7.37	-14.09±15.65	40.70±11.72	18.18±7.16	-4.21±15.74	19.45±7.39	-6.55±15.26	690.27±623.25	153.39±144.21	9.24±13.95	86.61±27.90	391.39±370.08	35.35±47.67	302.58±258.12	42.50±62.47
Mid-Holocene-CCSM	6.08±10.43	12.04±2.22	29.54±9.48	1105.73±450.68	27.31±6.84	-16.00±15.60	43.31±12.92	18.55±7.00	-6.14±15.67	19.85±6.79	-8.43±15.22	717.18±648.01	163.90±159.02	8.87±13.75	87.98±28.55	418.81±406.08	33.80±46.85	329.15±289.49	39.50±58.41
Mid-Holocene-MIROC	6.60±9.83	12.01±2.53	28.68±8.55	1127.64±462.29	28.61±6.46	-15.73±15.56	44.35±14.19	19.24±7.13	-5.57±15.33	20.87±6.65	-8.21±14.64	734.49±723.82	172.10±180.62	9.15±14.59	86.10±31.17	430.50±460.22	35.47±50.01	344.24±338.40	43.00±63.55
Mid-Holocene-MPI	6.26±10.30	12.02±2.28	29.67±9.56	1091.87±447.53	27.25±6.88	-15.86±15.59	43.08±13.07	18.50±7.02	-5.73±15.45	19.85±6.79	-8.14±15.14	769.64±720.16	179.99±186.21	8.77±13.59	89.14±29.19	450.01±450.38	34.30±47.72	349.77±310.25	40.21±61.89
Average Holocene	*6.31±0.26*	*12.02±0.02*	*29.30±0.54*	*1108.41±18.04*	*27.72±0.77*	*-15.91±0.16*	*43.58±0.68*	*18.76±0.41*	*-5.81±0.29*	*20.19±0.59*	*-8.26±0.15*	*740.44±26.73*	*172.00±8.05*	*8.93±0.20*	*87.74±1.53*	*433.11±15.76*	*34.52±0.86*	*341.05±10.67*	*40.90±1.85*
LGM-CCSM	3.17±11.47	15.77±5.29	37.24±11.31	980.67±415.09	24.13±8.01	-19.48±16.83	43.62±12.77	13.72±7.86	-7.70±16.53	15.10±7.97	-10.08±16.17	644.85±675.09	139.84±146.84	9.09±15.71	86.66±29.79	358.82±387.10	34.45±55.13	274.59±273.16	41.55±69.50
LGM-MIROC	4.12±11.83	11.75±2.84	31.13±9.68	980.59±414.91	23.07±7.94	-16.52±17.25	39.60±12.20	14.69±7.89	-6.80±16.9	16.05±8.23	-9.13±16.61	709.03±761.49	157.19±182.97	9.78±16.62	86.45±29.58	402.02±474.85	37.80±58.22	291.78±293.85	46.63±76.16
LGM-MPI	3.44±11.57	12.68±2.59	32.68±10.39	1009.39±454.61	22.95±7.64	-18.47±17.42	41.42±13.00	14.47±7.59	-7.47±17.22	15.66±7.78	-10.25±16.75	703.76±657.96	154.72±143.36	9.66±15.40	87.14±28.31	385.62±354.66	37.77±55.27	305.50±266.64	54.81±107.97
Average LGM	*3.58±0.49*	*13.40±2.10*	*33.68±3.18*	*990.22±16.60*	*23.38±0.65*	*-18.16±1.50*	*41.55±2.01*	*14.29±0.21*	*-7.32±0.47*	*15.60±0.48*	*-9.82±0.60*	*685.87±35.62*	*150.58±9.39*	*9.51±0.37*	*86.75±0.35*	*382.15±21.81*	*36.67±1.93*	*290.62±15.49*	*47.66±6.69*
2070-CCSM RCP 2.6	8.79±10.37	19.94±2.18	31.06±9.13	1007.92±411.17	28.24±7.56	-12.18±15.19	40.42±11.47	19.84±6.98	-2.42±15.45	21.13±7.27	-4.74±14.95	722.84±650.86	163.83±156.09	8.77±13.03	85.55±27.49	413.94±392.39	34.80±45.09	316.30±272.36	43.19±65.86
2070-GFDL RCP 2.6	10.44±10.11	11.79±2.45	30.38±9.34	1027.8±420.34	30.33±6.89	-10.51±14.89	40.85±11.60	21.60±6.83	-0.57±15.61	23.17±6.97	-3.23±14.72	777.33±666.59	182.03±162.82	9.53±14.03	87.33±26.87	452.31±403.21	38.14±50.21	345.84±282.00	47.36±68.83
2070-MPI RCP 2.6	8.82±10.59	11.84±2.20	31.01±9.28	992.12±406.15	28.04±7.46	-12.15±15.54	40.20±11.47	19.68±7.11	-2.25±15.92	20.98±7.47	-4.44±15.41	695.88±625.12	157.10±148.87	9.83±14.50	83.86±26.99	394.36±381.18	37.87±49.93	297.56±260.11	47.12±97.30
2070-CCSM RCP 8.5	11.22±10.04	11.79±2.10	30.63±9.01	1003.82±403.73	30.74±7.15	-9.65±14.71	40.39±11.19	22.05±6.93	3.78±15.00	23.57±7.13	-2.25±14.50	762.11±695.42	176.26±170.55	8.55±11.89	85.30±28.19	443.62±433.01	34.77±42.05	327.14±292.03	43.68±74.04
2070-GFDL RCP 8.5	13.02±9.71	11.66±2.54	30.30±9.54	1023.42±404.61	32.82±6.68	-7.58±14.01	40.40±10.92	24.16±6.60	2.50±15.06	25.77±6.74	-0.49±14.02	789.20±672.20	187.18±163.54	9.00±12.34	88.74±27.57	461.62±406.18	36.43±44.26	358.19±292.63	48.37±75.14
2070-MPI RCP 8.5	11.42±10.3	11.65±2.17	30.42±9.25	994.34±403.85	31.00±7.45	-9.33±15.21	40.33±11.37	21.93±7.29	0.94±15.76	23.76±7.43	-1.94±14.94	718.87±665.43	162.51±160.23	9.81±14.79	82.18±27.40	410.75±412.47	38.30±54.54	300.44±274.46	50.89±131.65
Average 2070	*10.62±1.64*	*13.11±3.35*	*30.63±0.33*	*1008.24±14.74*	*30.20±1.81*	*-10.23±1.77*	*40.43±0.22*	*21.54±1.65*	*0.33±2.53*	*23.06±1.80*	*-2.85±1.61*	*744.37±37.08*	*171.49±12.05*	*9.25±0.55*	*85.49±2.35*	*429.43±26.76*	*36.72±1.64*	*324.25±24.37*	*46.77±2.91*

Table S4. Mean values standard deviation for the 19 bioclimatic variables for *Davidia involucrata* occurrences. The mean values have been obtained averaging the individual values for all 275 species occurrences.

	**Variable**																		
**Climatic scenario**	bio1 (ºC)	bio2 (ºC)	bio3 (%)	bio4 (ºC × 100)	bio5 (ºC)	bio6 (ºC)	bio7 (ºC)	bio8 (ºC)	bio9 (ºC)	bio10 (ºC)	bio11 (ºC)	bio12 (mm)	bio13 (mm)	bio14 (mm)	bio15 (%)	bio16 (mm)	bio17 (mm)	bio18 (mm)	bio19 (mm)
Present	12.10±3.16	8.45±1.34	30.19±5.76	675.73±90.30	25.61±3.96	-2.15±3.47	27.76±2.07	19.52±3.42	3.05±2.83	20.42±3.80	3.00±2.82	1193.93±209.83	214.16±39.09	17.75±9.58	70.52±13.83	580.32±95.11	61.44±31.07	565.55±93.16	61.72±30.93
Mid-Holocene-CCSM	11.25±3.19	8.56±1.35	29.14±5.40	733.83±90.29	25.38±4.72	-3.71±3.57	29.10±2.19	20.04±3.78	1.46±2.83	20.20±3.80	1.38±2.86	1268.30±230.11	240.10±50.53	16.81±8.95	76.15±13.72	648.99±114.76	57.74±27.97	618.48±114.52	58.12±27.75
Mid-Holocene-MIROC	10.97±3.28	8.33±1.38	28.62±4.73	736.06±74.70	24.74±4.04	-3.88±3.83	28.66±2.09	19.51±3.61	1.22±2.98	19.90±3.82	1.17±2.92	1357.64±241.46	286.66±56.29	16.21±8.50	79.43±16.12	719.84±131.36	56.40±27.73	704.10±131.53	56.54±27.70
Mid-Holocene-MPI	10.85±3.17	8.10±1.32	27.62±4.86	745.81±76.37	24.86±3.90	-4.04±3.49	28.90±1.85	19.71±3.68	1.15±2.68	20.05±3.77	0.96±2.74	1278.55±215.60	231.57±47.42	15.12±6.60	72.87±10.79	631.98±109.06	60.37±24.10	614.13±109.36	60.68±23.87
Average Holocene	11.02±0.21	8.33±0.23	28.46±0.77	738.57±6.37	24.99±0.34	-3.88±0.17	28.89±0.22	19.75±0.27	1.28±0.16	20.05±0.15	1.17±0.21	1301.50±48.89	252.78±29.65	16.05±0.86	76.15±3.28	666.94±46.60	58.17±2.02	645.57±50.74	58.45±2.09
LGM-CCSM	7.13±2.98	9.09±1.37	31.81±5.27	670.32±90.27	20.96±3.73	-7.36±3.31	28.32±2.42	14.52±3.24	-1.88±2.86	15.43±3.49	-1.93±2.83	1408.13±360.22	250.18±54.17	21.24±12.83	70.90±13.91	679.78±147.14	73.16±41.63	661.78±142.27	73.43±41.45
LGM-MIROC	8.98±3.18	8.35±2.02	29.91±7.39	670.77±90.37	22.45±3.80	-5.13±3.60	27.58±1.91	16.38±3.39	-0.02±2.93	17.29±3.72	-0.07±2.93	1437.94±423.71	253.52±54.98	22.06±14.25	70.89±13.90	689.05±153.52	76.10±46.91	670.88±147.46	76.47±46.79
LGM-MPI	8.25±3.27	8.66±1.32	30.44±5.10	666.50±78.18	21.70±4.05	-6.45±3.67	28.15±2.16	15.61±3.52	-0.88±2.86	16.47±3.85	-0.91±2.89	1174.32±182.00	211.09±48.11	19.74±10.30	68.57±13.47	555.42±95.23	68.65±30.74	543.52±93.54	68.92±30.87
Average LGM	8.12±0.93	8.70±0.37	30.72±0.98	669.20±2.35	21.70±0.75	-6.31±1.12	28.02±0.39	15.50±0.93	-0.93±0.93	16.40±0.93	-0.97±0.93	1340.13±144.37	238.26±23.59	21.01±1.18	70.12±1.34	641.42±74.62	72.64±3.75	625.39±71.05	72.94±3.80
2070-CCSM RCP 2.6	13.39±3.13	8.58±1.26	30.65±5.44	670.10±90.04	26.87±3.94	-0.83±3.37	27.70±1.98	20.81±3.42	4.45±2.78	21.75±3.79	4.40±2.78	1242.34±206.69	230.55±44.46	15.97±8.19	73.35±12.04	623.07±108.29	57.82±28.36	604.95±107.01	58.18±28.15
2070-GFDL RCP 2.6	15.18±3.13	8.68±1.28	31.05±5.69	686.42±101.38	29.04±4.00	1.29±3.30	27.74±2.31	22.60±3.63	6.08±2.74	23.74±3.88	6.03±2.76	1180.37±227.35	222.15±46.66	17.06±8.70	69.67±7.76	568.31±97.71	65.20±30.85	552.86±99.52	65.91±30.40
2070-MPI RCP 2.6	13.64±3.13	8.64±1.30	31.10±5.99	654.80±97.00	27.14±4.12	-0.50±3.39	27.64±2.35	20.75±3.44	4.90±2.82	21.78±3.80	4.86±2.81	1171.58±198.17	219.32±40.24	17.21±10.03	70.70±15.86	575.47±101.28	61.26±31.42	554.00±103.57	61.87±31.11
2070-CCSM RCP 8.5	15.61±3.14	8.21±1.10	29.78±5.25	685.47±93.23	29.16±4.05	1.82±3.22	27.33±2.04	23.52±3.54	6.52±2.74	24.05±3.83	6.47±2.73	1253.10±207.90	232.13±39.97	13.00±5.21	74.25±9.98	628.64±103.06	49.42±19.90	617.80±106.32	49.83±19.70
2070-GFDL RCP 8.5	17.00±3.13	9.06±1.21	31.10±5.16	719.15±96.71	31.12±3.95	2.28±3.26	28.83±2.41	25.48±3.88	9.32±3.40	25.87±3.81	7.39±2.70	1126.87±220.45	216.16±45.14	16.82±8.96	74.69±4.49	573.92±112.35	61.10±23.92	557.81±118.20	66.61±29.26
2070-MPI RCP 8.5	15.32±3.11	8.81±1.30	30.78±5.91	674.94±95.45	29.84±4.06	1.35±3.32	28.48±2.32	23.13±3.39	6.97±2.77	24.37±3.81	6.94±2.76	1188.93±217.26	225.74±39.90	15.41±9.54	74.48±14.32	601.86±105.27	53.89±29.74	576.92±101.72	54.27±29.45
Average 2070	15.02±1.34	8.66±0.28	30.74±0.51	681.81±21.65	28.86±1.62	0.90±1.27	27.95±	22.72±0.57	6.37±1.73	23.59±1.59	6.02±1.17	1193.87±47.01	224.34±6.29	15.91±1.59	72.86±2.14	595.21±26.47	58.12±5.70	577.39±28.02	59.45±6.63

Table S5. The suitable areas (potential habitats) included within (and outside) the network of protected areas (PAs) (nature reserves) in China.

	Total Predicted area in China (km^2)	Total predicted area in China within PAs (km^2 and %)	Total predicted area in China outside PAs (km^2 and %)	Predicted area in China: threshold-0.5 China (km^2)	Predicted area in China (threshold-0.5) within PAs (km^2 and %)	Predicted area in China (threshold-0.5) outside PAs (km^2 and %)	Predicted area in China: 0.5-1 (km^2)	Predicted area in China (0.5-1) within PAs (km^2 and %)	Predicted area in China (0.5-1) outside PAs (km^2 and %)
Present	511,468	133,409 (26.08)	378,059 (73.92)	328,718	77,205 (23.49)	251,513 (76.51)	182,750	56,204 (30.75)	126,546 (69.25)
2070-CCSM RCP 2.6	339,827	104,203 (30.66)	235,624 (69.34)	207,021	60,189 (29.07)	146,832 (70.93)	132,806	44,014 (33.14)	88,792 (66.86)
2070-GFDL RCP 2.6	179,024	61,043 (34.10)	117,981 (65.90)	142,677	45,025 (31.56)	97,652 (68.44)	36,347	16,018 (44.07)	20,329 (55.93)
2070-MPI RCP 2.6	342,958	102,318 (29.83)	240,640 (70.17)	252,436	71,057 (28.15)	181,379 (71.85)	90,522	31,261 (34.53)	59,261 (65.47)
2070-CCSM RCP 8.5	206,154	74,171 (35.98)	131,983 (64.02)	127,400	43,041 (33.78)	84,359 (66.22)	78,754	31,130 (39.53)	47,624 (60.47)
2070-GFDL RCP 8.5	93,521	39,682 (42.43)	53,839 (57.57)	77,039	32,158 (41.74)	44,881 (58.26)	16,482	7,524 (45.65)	8,958 (54.35)
2070-MPI RCP 8.5	133,324	50,911 (38.19)	82,413 (61.81)	96,609	34,751 (35.97)	61,858 (64.03)	36,715	16,160 (44.01)	20,555 (55.99)
Average 2070	215,801	72,055 (35.20)	143,747 (64.80)	150,530	47,704 (33.38)	102,827 (66.62)	65,271	24,351 (40.16)	40,920 (59.84)

Figure S1. Species distribution modeling in *Davidia involucrata* taking into account the uncertainty derived from the evaluation of model performance. The maps on the left are the "uncertainty" maps. The maps in the center are the "standard" models. The maps on the right are the "refined" models. (A-C) Present, (D-F) Mid-Holocene-CCSM, (G-I) Mid-Holocene-MIROC, (J-L) Mid-Holocene-MPI, (M-O) LGM-CCSM, (P-R). LGM-MIROC, (S-U) LGM-MPI, (V-X) 2070-CCSM RCP 2.6, (Y-Za) 2070-GFDL RCP 2.6, (Zb-Zd) 2070-MPI RCP 2.6, (Ze-Zg) 2070-CCSM RCP 8.5, (Zh-Zj) 2070-GFDL RCP 8.5, (Zk-Zm), 2070-MPI RCP 8.5. Purple lines: national boundaries between China and India (at issue). Maps were generated using the software ArcGIS 10.2.

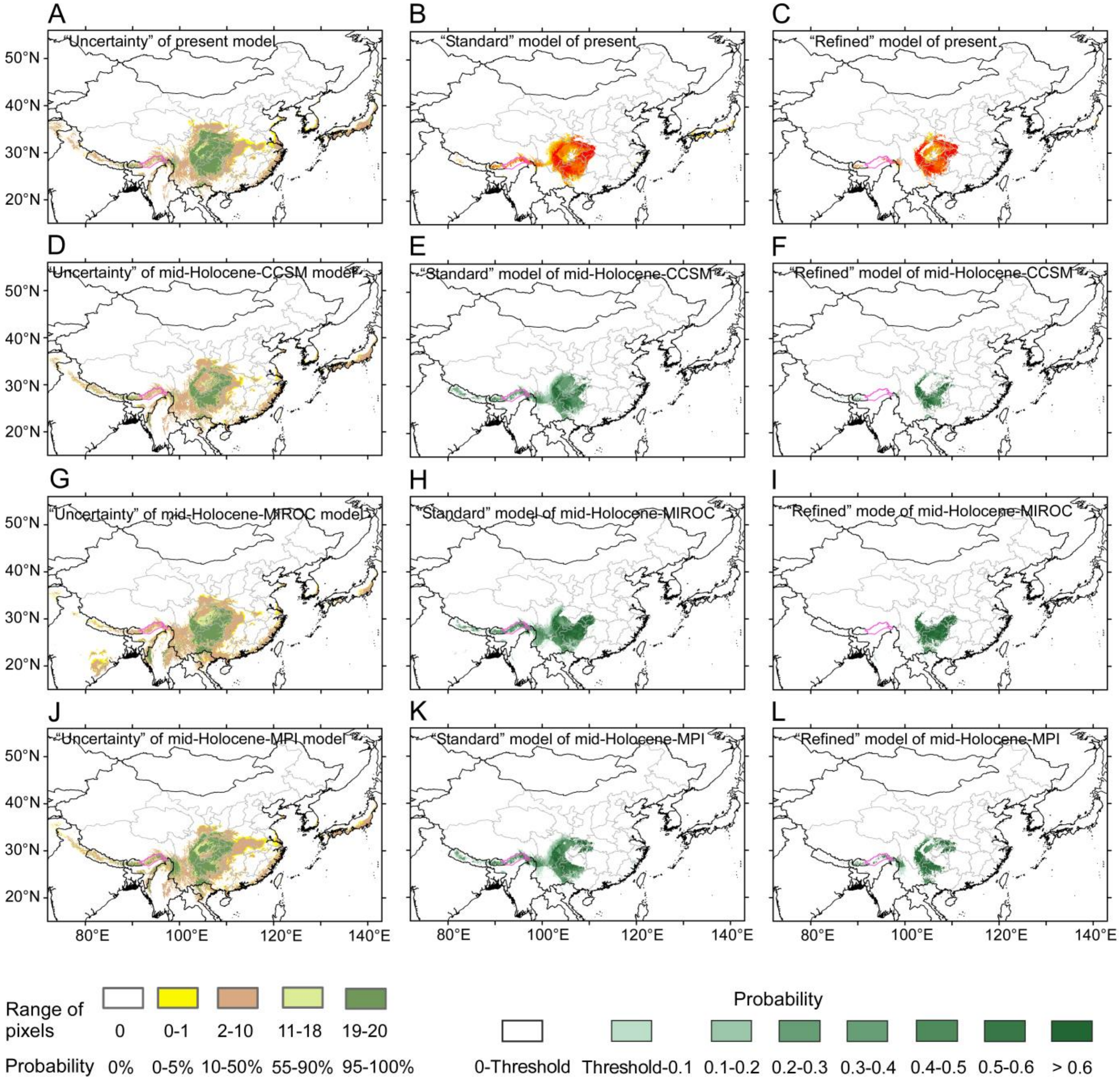

Figure S1 Continued.

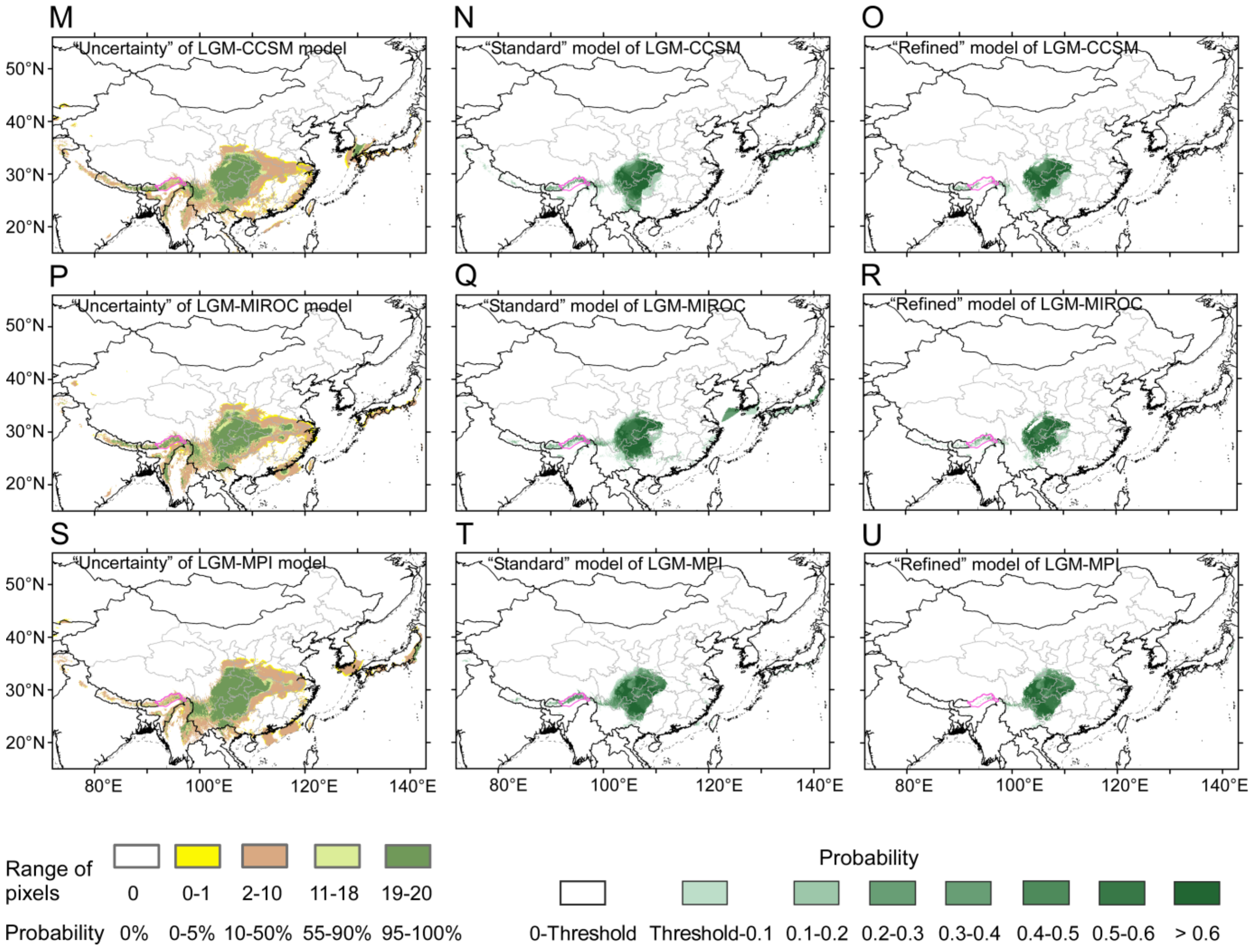

Figure S1 Continued.

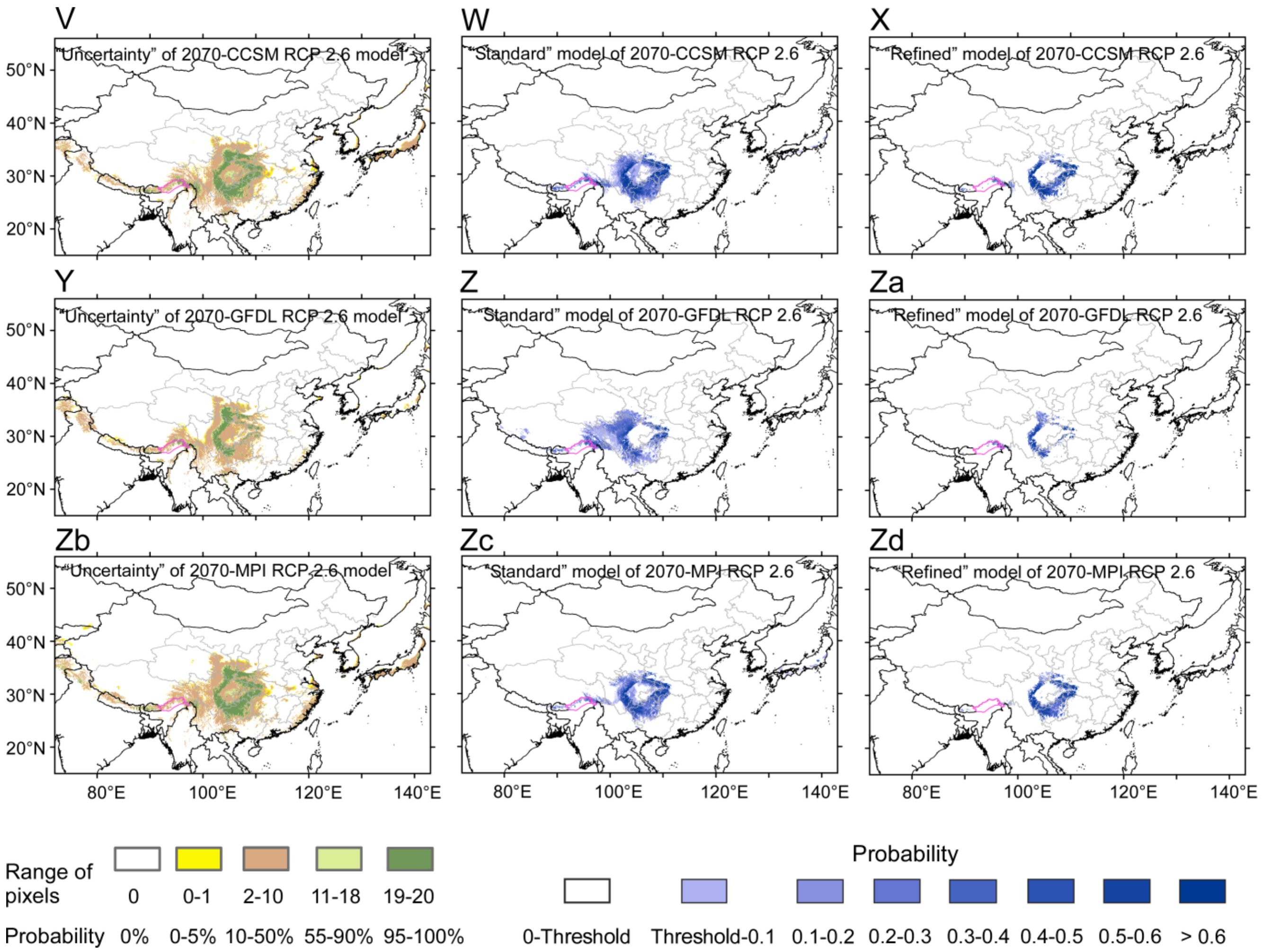

Figure S1 Continued.

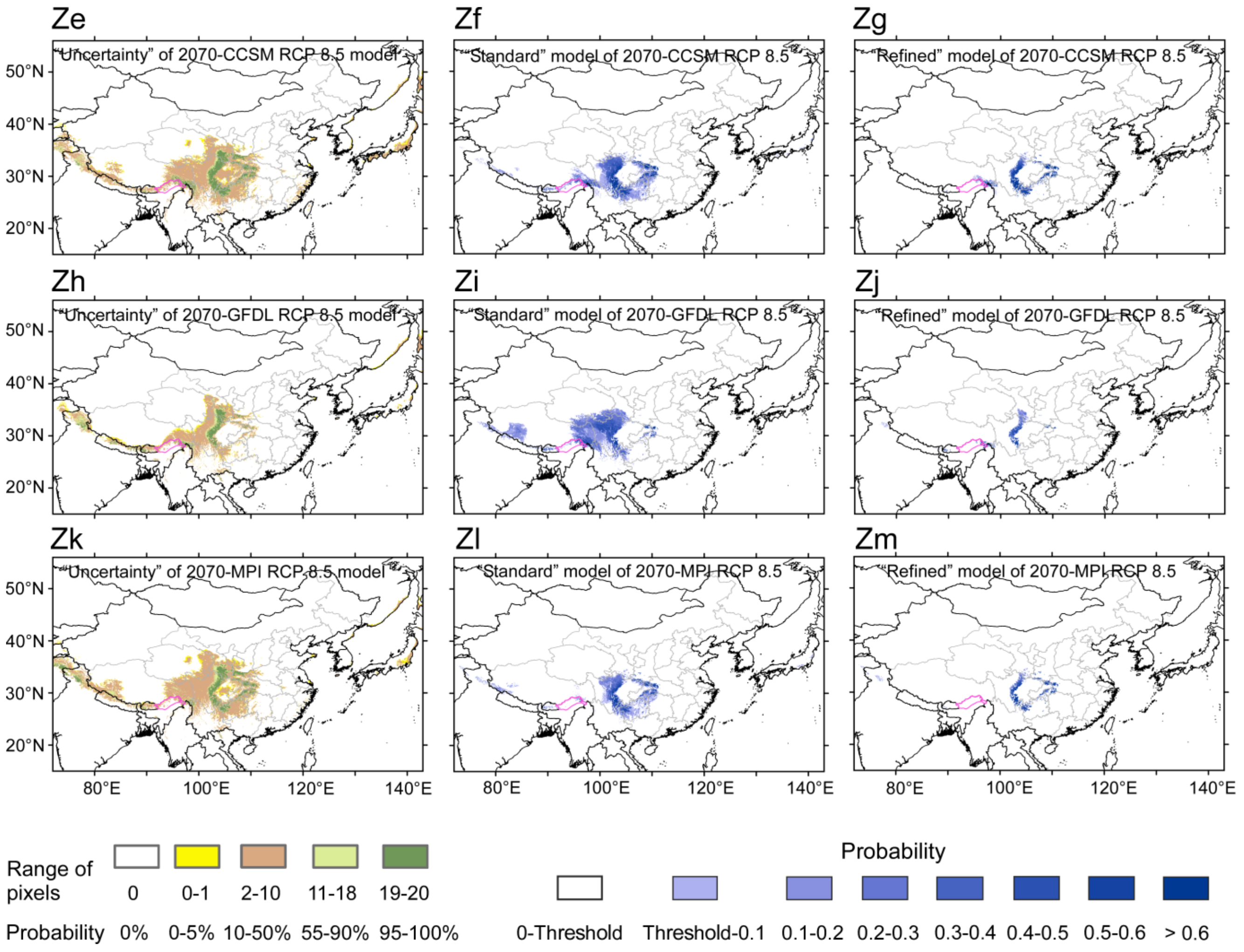

Figure S2. Mean and standard deviation (SD) values for four bioclimatic variables (bio1, annual mean temperature in ºC; bio4, temperature seasonality (SD × 100); bio12, annual precipitation in mm; bio18, precipitation of the warmest quarter) considering different periods (LGM, Holocene, present and 2070) for: (A), (B), (C) and (D) all the 2.5 arc-min cells contained within the study area 1,239,717 cells; (E), (F), (G) and (H) all 275 occurrences of the species.

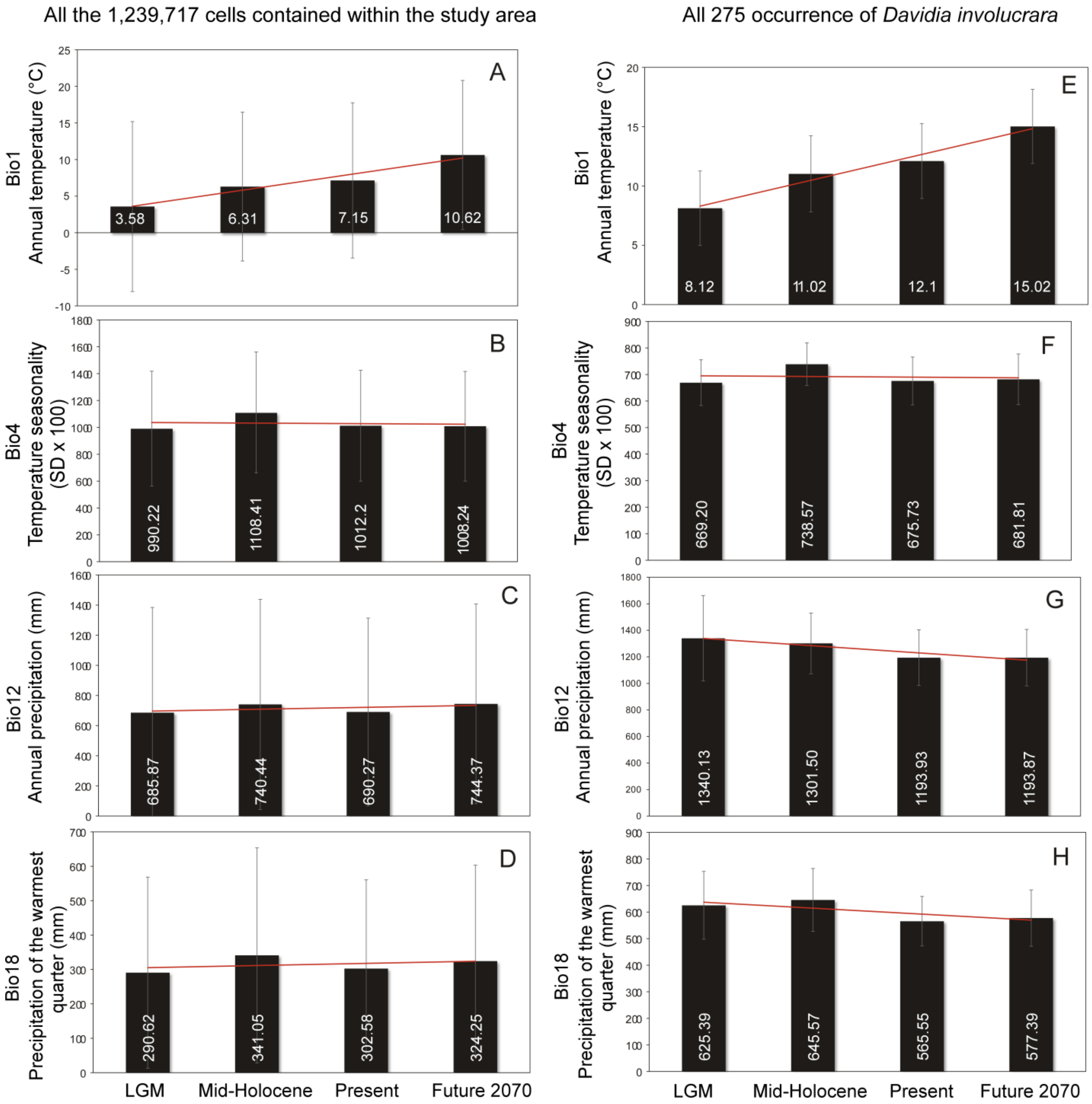

Figure S3. Predicted potential habitats with and without protection in China under future 2070 climatic scenarios, with areas proposed for conservation. (A) 2070-GFDL RCP 2.6, (B) 2070-MPI RCP 2.6, (C) 2070-CCSM RCP 8.5, (D) 2070-GFDL RCP 8.5, (E) 2070-MPI RCP 8.5. Purple lines: national boundaries between China and India (at issue). Maps were generated using the software ArcGIS 10.2.

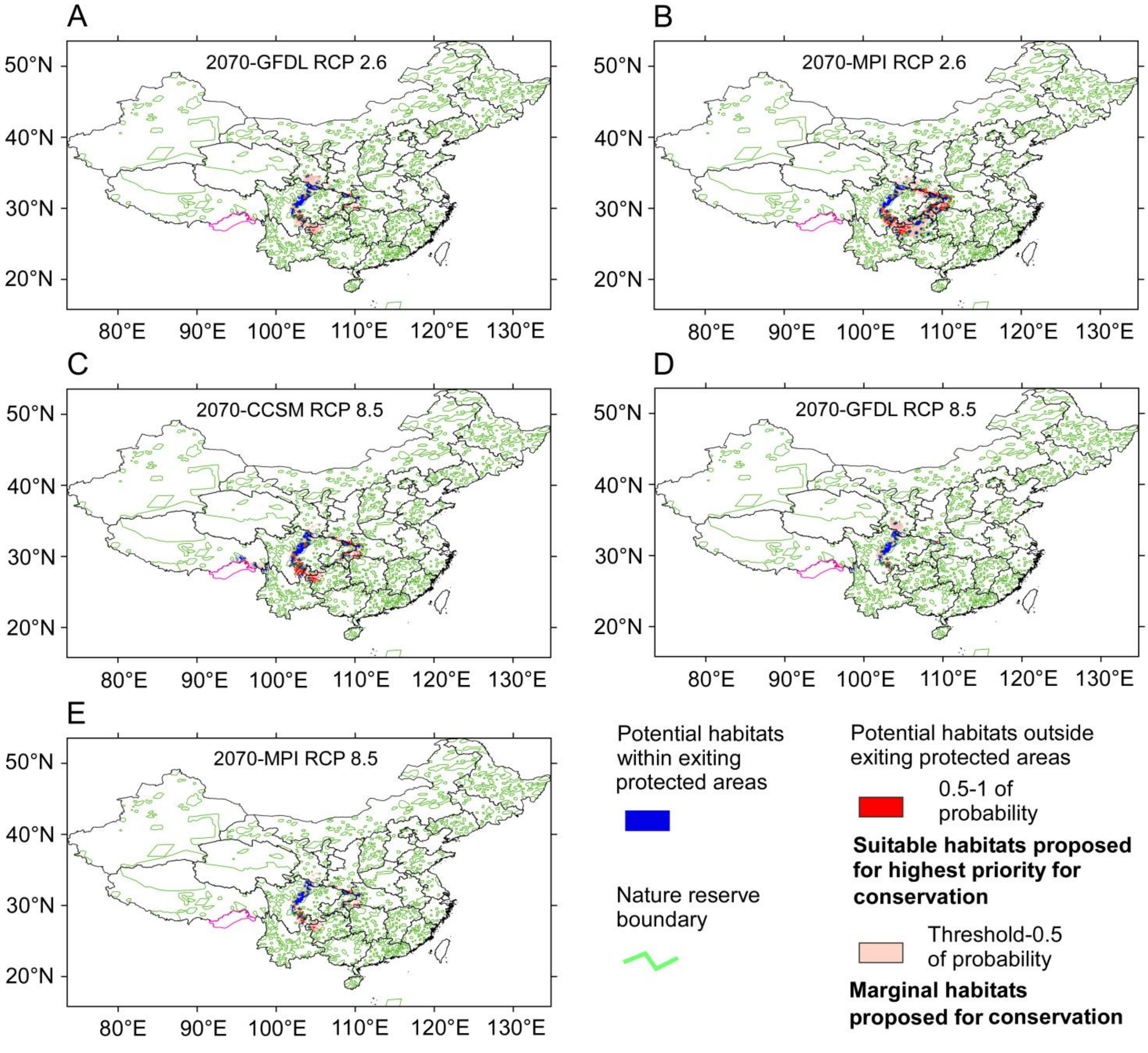

Supplement: Supplementary Information [file srep43822-s1.pdf]
